# Synthesis and Relaxivity Studies of a DOTA-Based Nanomolecular Chelator Assembly Supported by an Icosahedral *Closo*-B_12_^2−^ -Core for MRI: A Click Chemistry Approach 

**DOI:** 10.3390/molecules18089034

**Published:** 2013-07-29

**Authors:** Lalit N. Goswami, Lixin Ma, Peter J. Kueffer, Satish S. Jalisatgi, M. Frederick Hawthorne

**Affiliations:** International Institute of Nano and Molecular Medicine, School of Medicine, University of Missouri, Columbia, MO 65211-3450, USA

**Keywords:** click reaction, *closo*-boranes, MRI contrast agents, relaxivity, gadolinium complexes, DOTA

## Abstract

An icosahedral *closo-*B_12_^2−^ scaffold based nano-sized assembly capable of carrying a high payload of Gd^3+^-chelates in a sterically crowded configuration is developed by employing the azide-alkyne click reaction. The twelve copies of DO3A-*t*-Bu-ester ligands were covalently attached to an icosahedral *closo-*B_12_^2−^ core via suitable linkers through click reaction. This nanomolecular structure supporting a high payload of Gd^3+^-chelate is a new member of the closomer MRI contrast agents that we are currently developing in our laboratory. The per Gd ion relaxivity (*r*_1_) of the newly synthesized MRI contrast agent was obtained in PBS, 2% tween/PBS and bovine calf serum using a 7 Tesla micro MRI instrument and was found to be slightly higher (*r*_1_ = 4.7 in PBS at 25 °C) compared to the clinically used MRI contrast agents Omniscan (*r*_1_ = 4.2 in PBS at 25 °C) and ProHance (*r*_1_ = 3.1 in PBS at 25 °C).

## 1. Introduction

**Figure 1:**
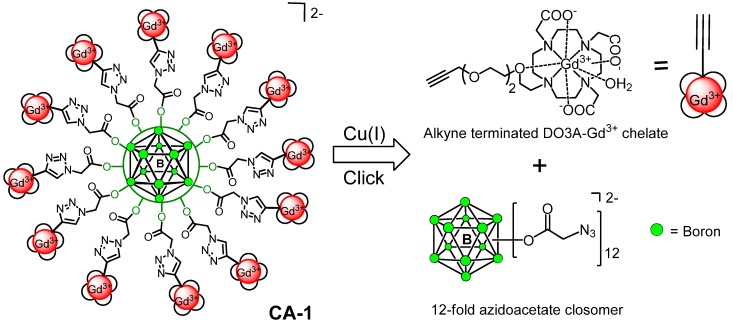
Schematic representation of click assisted synthesis of a [*closo*-B_12_]^2−^ scaffold supporting 12 copies of a Gd^3+^-DOTA chelate.

Magnetic Resonance Imaging (MRI) is one of the most successful non-invasive diagnostic imaging techniques used in medicine [1,2]. Image contrast in MRI depends principally on differences in the relaxation times and proton density provided by water among neighboring tissues. The contrast between adjacent tissues can be altered by the administration of an MRI contrast agent (CA). The effectiveness of a CA is measured by the relaxivity *r*_1_ [mM^−1^s^−1^], which represents the increase in the water proton relaxation rate R1 in presence of the CA. The CAs used in clinical MRI are species that usually contain a paramagnetic metal ion, such as gadolinium (Gd^3+^) chelated within a diethylenetriaminepentaacetic acid (DTPA) or 1,4,7,10-tetraazacyclododecane- 1,4,7,10-tetraacetic acid (DOTA) ligands [3]. The non-targeted MRI CAs commonly used in clinical settings are small molecular weight compounds and have some disadvantages, namely relatively low *r*_1_ values (~3–4 mM^−1^s^−1^) at higher magnetic field strength, nephrogenic systemic fibrosis (NSF) in patients with renal dysfunction, and extremely short intravascular half-life (~20 min) [4,5]. According to the Solomon-Bloembergen-Morgan (SBM) theory [6,7], the relaxivity of Gd^3+^-based CAs can be improved by reducing their rotational motion in solution, e.g., by immobilizing the gadolinium complexes onto macromolecules of different shapes and sizes (proteins, polylysine, dendrimers, polysaccharides, micelles, liposomes *etc.*). The macromolecular CAs carrying multiple copies of Gd^3+^ chelates has shown great promise for enhancing the contrast, sensitivity and diagnostic imaging time frame, as well as slow vascular diffusion and clearance rates due to the enhanced permeability and retention (EPR) effect [8,9,10,11,12,13,14,15]. We herein report the click chemistry assisted synthesis and relaxivity measurements of a monodisperse, nanomolecular polyfunctional MRI CA that contains twelve radial Gd^3+^-DOTA chelate arms in close proximity, linked to a central rigid *closo*-B_12_^2−^ core via a suitable linker (**CA-1**, Figure 1). 

## 2. Results and Discussion

The icosahedral dodecahydro-*closo*-dodecaborate [*closo*-B_12_H_12_]^2−^ ion contains 12 identical vertices, each of which can be attached to pre-determined identical or differing substituents to generate attractive molecular construction modules. The major breakthrough towards the 12-fold functionalization of [*closo*-B_12_H_12_]^2−^ ion came with the discovery of the B-H hydroxylation reaction which led to a robust synthesis of [*closo*-B_12_(OH)_12_]^2−^ (**2**) molecular scaffold that can be used to anchor up to 12 radial arms with desired pendant groups even at the generation zero [16]. The chemistry of the icosahedral [*closo*-B_12_(OH)_12_]^2−^ ion is broadening into a nanoparticle-size molecular architecture useful for carrying large payloads of pharmaceuticals or imaging agents. This is possible due to the facile functionalization of the B-OH vertices in essentially the same fashion as for alcohols. Subsequently, 12-fold ester, carbamate and ether derivatives, described by us as “*closomers*” are reported [17,18,19]. This unique platform provides an unprecedented multi-centric core for the development of high payload delivery systems for the pharmaceuticals and imaging agents. The 12 vertices of [*closo*-B_12_(OH)_12_]^2−^ can be further branched to generate dendritic closomers [20]. However, compared to the dendrimers of similar functionality, the closomer derivatives of [*closo*-B_12_(OH)_12_]^2−^ are monodispersed, compact molecules having greater rigidity and symmetry. These unique characteristics of closomers are ideally suited for the development of high performance macromolecular CAs [21]. 

The Cu(I)- catalyzed [3+2] cycloaddition of an alkyne and an azide functionality to generate five membered 1,2,3- triazole rings, a hallmark reaction in “Click” chemistry has found immense applications in biomedical research and material science [22,23] due to its very efficient and high yielding product formation. One of the requirements for the development of closomer chemistry for drug delivery application is availability of a high-yielding, multi-vertex coupling reaction such as click reaction. Therefore, blending click and closomer chemistries offers immense opportunities towards formation of novel drug molecules and imaging entities. We recently reported, for the first time, the use of 12-fold click reaction on closomer surface for the construction of discrete nano-size molecules carrying multiple copies of diethylenetriaminetetraacetic acid (DTTA)-Gd^3+^ chelates for MRI applications [24]. However, the urgency to pursue work presented here using DOTA type ligands was based on the fact that eight coordinate Gd^3+^-DOTA chelates has shown higher thermodynamic stability (log K_GdL_~23) compared to the seven coordinate Gd^3+^-DTTA chelates (log K_GdL_~17–19). Despite of high relaxivity value, *r*_1_ = 13.8 in PBS at 25 °C [24], the Gd^3+^-DTTA chelates are unfit for safe human use due to their compromised thermodynamic stability [1].

### Synthesis of 12-Fold Azidoacetate Closomer **4**

**Scheme 1:**
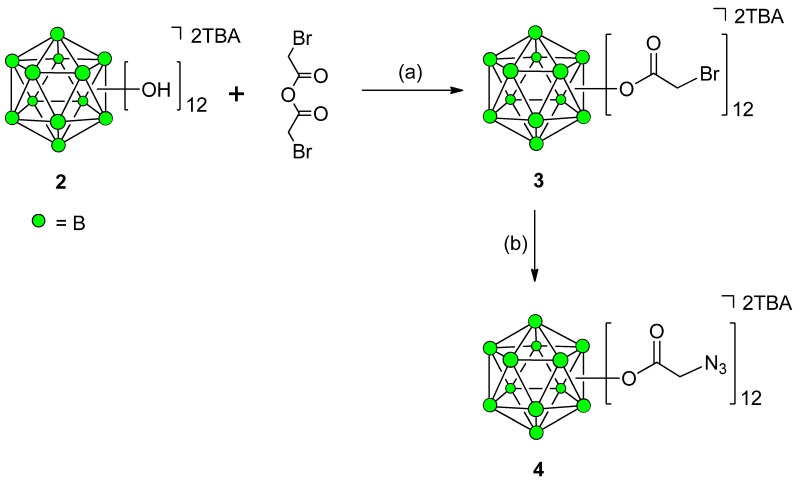
Synthesis of 12-fold azidoacetate closomer **4**.

Previously, we have synthesized 12-fold azidoacetate closomer **4** via 12-fold esterification of TBA_2_-**2** with chloroacetic anhydride followed by displacement of chloro functions with azido groups by reacting with NaN_3_ to form the 12-fold azidoacetate closomer **4**. The presence of twelve azide groups on a single, compact *closo*-borane cage provides us with the opportunity to perform 12-fold click reactions with adequately functionalized terminal alkyne moieties, thus leading to the synthesis of novel 1,2,3-triazole ring attached polyfunctional closomers entities [19]. In this article, we describe an improved procedure for the synthesis of 12-azidoacetate closomers **4** via 12-fold bromoacetate closomer **3**. Replacing the α-chloro with a more labile bromo leaving group provided more efficient substitution with azido group and a shorter reaction times. Under similar reaction conditions as those used to prepare 12-fold chloroacetate closomer [19], the TBA_2_-**2** and bromoacetic anhydride (5.0 eq. per vertices) were heated at reflux temperature in acetonitrile (ACN) for 5 days resulting in the formation of 12-fold bromoacetate closomer **3** in 79% yield after purification using size-exclusion chromatography on a Lipophilic Sephadex LH-20 column (Scheme 1). The closomer **3** was reacted with a 10 folds excess of sodium azide in dimethylformamide (DMF) at room temperature (RT) and as expected, the reaction was completed in 2 days to give the 12-fold azidoacetate closomer **4** in 88% yield (Scheme 1). 

*Reagents and Conditions*: (**a**) ACN, 5 days, reflux, 79%; (**b**) NaN_3_, DMF, 2 days, RT, 88%.

### Synthesis of DO3A Ligands

**Scheme 2:**
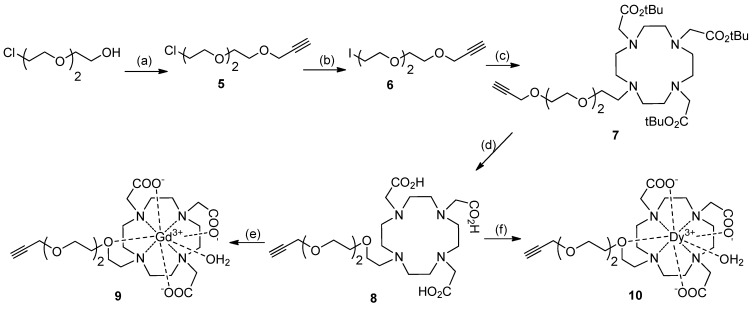
Synthesis of alkyne-terminated DO3A ligands.

We synthesized an alkyne terminated DOTA based ligand **7** from commercially available DO3A-*t*-Bu-ester (Scheme 2). First, a heterobifunctionalized alkyne-terminated short PEG linker, **6**, that has an Iodo- group at the distal end, was synthesized from commercially available 2-[2-(2-chloroethoxy)ethoxy]ethanol [25]. The reaction of **6** with DO3A-*t*-Bu-ester afforded DO3A-*t*-Bu-ester ligand **7** in 94% yield. The *tert*-butyl groups on **7** were then deprotected using formic acid to obtain the alkyne terminated DO3A derivative, **8**, in quantitative yield (95%). The Gd^3+^ complex **9** was synthesized by reacting **8** with Gd_2_O_3_ in water at 100 °C for 12 h. Similarly, the Dy^3+^-complex of the DO3A ligand, **10**, was synthesized in 92% yield by reacting **8** with DyCl_3_·6H_2_O in pyridine (Scheme 2).

### Synthesis of Closomer Contrast Agents CA-1

**Scheme 3:**
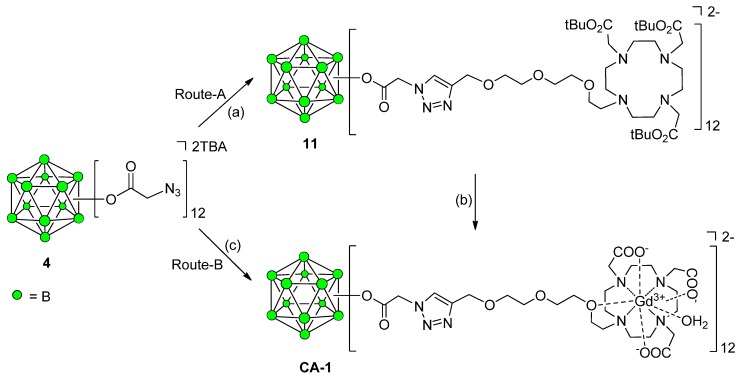
Synthesis of closomer contrast agent **CA-1**.

The closomer-DO3A conjugate **11** was synthesized in 67% yield by reacting **4** with the alkyne-terminated DO3A ligand **7** under Cu(I)-catalyzed 1,3-dipolar cycloaddition reaction conditions. The closomer **11** was purified by size-exclusion column chromatography using Lipophilic Sephadex LH-20 using ACN as the eluent and characterized by IR, NMR and HRMS spectroscopy. Closomer **11** exhibited a characteristic singlet at δ 7.74 ppm in the ^1^H-NMR spectrum that was assigned to the 12 alkene-*CH* protons of the 12 triazole rings. The IR spectrum of the product did not exhibit the characteristic peak at 2109 cm^−1^, which was attributed in closomer **4** to the asymmetric stretching of the azide group (Supplementary Information or SI). Next, closomer **11** was treated with 80% trifluoroacetic acid (TFA) in dichloromethane (DCM) to remove the *tert*-butyl ester groups; complete deprotection was confirmed by the absence of a large singlet at δ 1.46 ppm in the ^1^H-NMR spectrum originally attributed to the 36 *tert*-butyl ester groups of the 12 DO3A ligands attached to the *closo*-B_12_^2−^-core in **11**. Subsequently, the deprotected closomer **11** was reacted with GdCl_3_·6H_2_O in citrate buffer at pH ~7 to obtain **CA-1** in 75% yield (Scheme 3, Route-A). The **CA-1** was purified via exhaustive dialysis in ultrapure water and was characterized using IR and HRMS spectroscopy. The IR spectrum of **CA-1** exhibited the characteristic shift of the carbonyl stretch from 1727 cm^−1^ to 1601 cm^−1^, which demonstrates the complexation of Gd^3+^ ions with carboxylic acid groups of the DO3A ligand (SI). The purity of **CA-1** was tested by size-exclusion HPLC (SE-HPLC) analysis, and the gadolinium loading was determined using inductively coupled plasma optical emission spectroscopy (ICP-OES), which showed the formation of fully loaded chelates with an average of 12 Gd^3+^ ions per closomer. The presence of any free Gd^3+^ ions in closomer contrast agent **CA-1** was tested by spectrophotometric method using xylenol orange [26], and found to contain no free Gd^3+^ ions. Alternatively, the synthesis of closomer contrast agent **CA-1** was also attempted via a pre-complexation route as shown in Scheme 3, Route-B using Cu(I)-catalyzed 1,3-dipolar cycloaddition reaction between the azidoacetate closomer **4** and alkyne-terminated DO3A-Gd^3+^ complex **9**. However, this route resulted in the lower yield (38%) of the **CA-1** as compared to the Route-A (71%, overall yield from **4**) shown in Scheme 3.

*Reagents and conditions*: (**a**) NaH, THF, 0 °C-RT-60 °C, 24 h, 80%; (**b**) NaI, acetone, 60 °C, 12 h, 95%; (**c**) DO3A-*t*-Bu-ester, KHCO_3_, DMF, 12 h, 60 °C, 94%; (**d**) HCOOH, 12 h, 65 °C, 95%; (**e**) Gd_2_O_3_, H_2_O, 12 h, 100 °C, 91%; (**f**) DyCl_3_.6H_2_O, pyridine, 70 °C, 12 h, 92%.

*Reagents and conditions*: (**a**) DO3A ligand **7**, CuI, DIPEA, ACN-THF, 5 days, RT, 67%; (**b**) (i) 80% TFA-DCM, 6 h, RT; (ii) GdCl_3_·6H_2_O, citrate buffer, pH 7, 20 h, RT, sonication; (iii) Dialysis, 1,000 MWCO, 48 h; (iv) Lyophilization, 75%; (**c**) **9**, CuSO_4_·5H_2_O, sodium ascorbate, ACN-H_2_O, 3 days, 60 °C, size-exclusion chromatography, 38%. Tetrabutylammonium (TBA), N, N-Diisopropylethylamine (DIPEA); Molecular weight cut-off (MWCO)

### Determination of Hydration Number (q)

**Figure 2:**
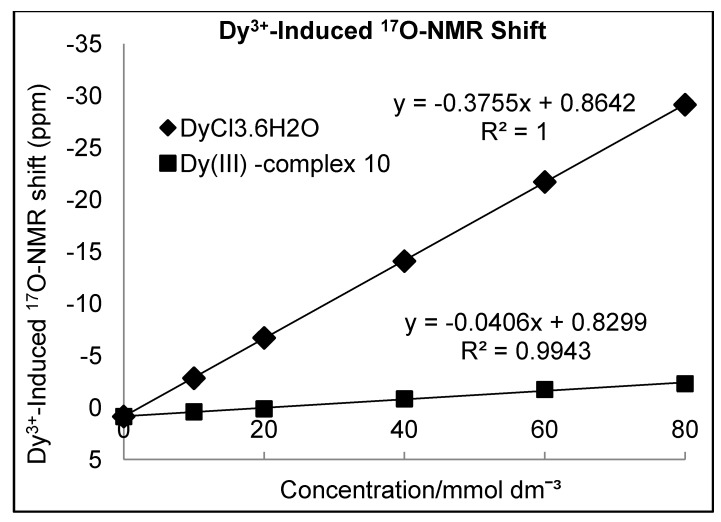
Plot of the Dy^3+^-induced water ^17^O-NMR shift as a function of [Dy].

In general, the *q* value of the Gd^3+^ complex of DOTA is predicted to be 1; however, the PEG oxygen donor in the modified DO3A ligand can partially displace the water molecules coordinated to the DO3A-Gd^3+^, **9**, consequently reducing the overall relaxivity of the CA [27]. In principle, the Gd^3+^-induced water ^17^O-NMR chemical shift can be used directly to determine the value of q, provided that the exchange between Gd^3+^ bound water and bulk water is fast on the ^17^O-NMR time scale. However, this situation exists at a temperature of about 80 °C. Furthermore, the severe line-broadening often makes it difficult to accurately measure q value on Gd^3+^ complexes and, therefore, measurements on the corresponding Dy^3+^ complex are preferred. Thus, the *q* value for the newly synthesized DO3A complex **9** was determined using the Dy^3+^-induced water ^17^O-NMR shifts (d.i.s.) method [28]. Various concentrations of Dy^3+^ complex **10** and DyCl_3_·6H_2_O over the range of 10-80 mmol dm^−3^ were prepared in 80% D_2_O-H_2_O and the d.i.s. (Δδ) was measured using a 400 MHz NMR instrument (Figure 2). The Δδ for a complex with the general formula Dy(ligand)_n_(H_2_O)_q_ is given by the relation (Δδ) = qΔ[Dy(ligand)_n_(H_2_O)_q_]/[H_2_O]. The slope of a plot of the d.i.s. *versus* the Dy^3+^ concentration is proportional to the *q* value of the complex. As expected the d.i.s. method gave a *q* value of 1 for the complex **10 **(SI). 

### Relaxivity Studies

The per-Gd relaxivity (*r*_1_) of closomer contrast agent **CA-1, **DO3A Gd^3+^-complex **9** and clinically used CA, Omniscan at 25 °C and 7 Tesla (T) were compared in three different formulations: PBS, 2% tween/PBS, and bovine calf serum. The relaxivity of each CA was relatively unaffected by formulation type, but the *r*_1_ values of **CA-1** (4.7 mM^−1^s^−1^ in PBS) were slightly higher than Omniscan (4.2 mM^−1^s^−1^ in PBS) and the DO3A Gd^3+^-complex **9** across all formulations (SI). Furthermore, these relaxivity values are comparable to our previously reported results [21]. The limited increase in *r*_1_ at 7 T can be attributed to the fact that the contribution of the slow tumbling (rotational correlation time or τ_R_) towards the enhancement of *r*_1_ is not observed at higher magnetic field strengths (4.7 T and above) [29].

**Figure 3:**
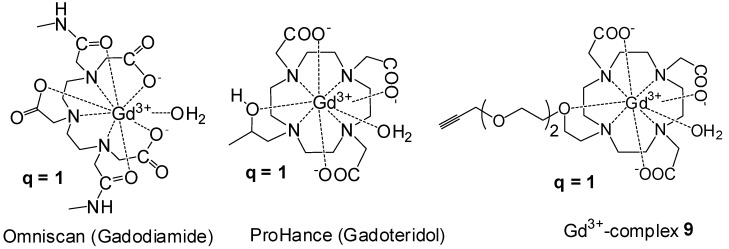
Structures of clinically used MRI CAs; Omniscan and ProHance and Gd^3+^-complex **9**.

**Figure 4:**
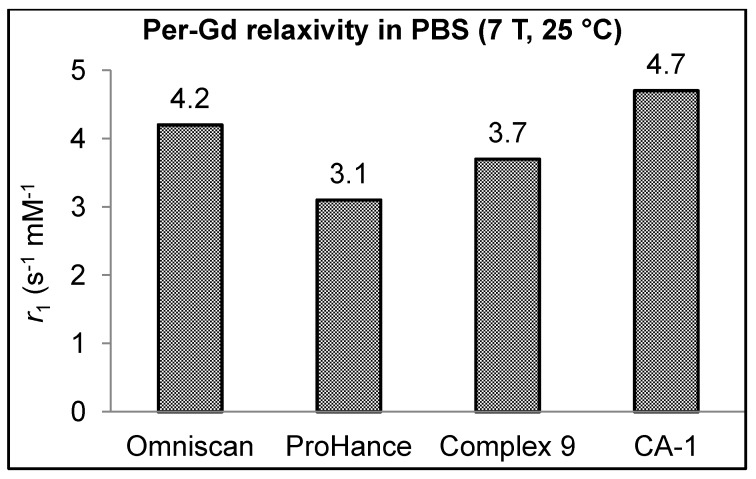
Comparison of the per-Gd *r*_1_ values of **CA-1**, DO3A Gd^3+^-complex **9** with clinically used CAs; Omniscan and ProHance.

Since Omniscan is a DTPA based ligand (acyclic polyaminocarboxylate core) that inherently has slightly higher *r*_1_ as compared to DOTA based ligands (cyclic polyaminocarboxylate core), we also compared the per-Gd *r*_1_ of DO3A Gd^3+^-complex **9** and closomer contrast agent **CA-1** with structurally similar clinically used MRI CA, ProHance shown in Figure 3 [30]. The macromolecular **CA-1** shows significantly higher *r*_1_ value (per Gd *r*_1_ 4.7 mM^−1^s^−1^; molecular *r*_1_ 56.5 mM^−1^s^−1^) as compared to ProHance (both per Gd and per molecular *r*_1_ 3.1 mM^−1^s^−1^) at 7 T, 25 °C in PBS (Figure 4). Macromolecular CAs such as **CA-1** are known to exhibit higher *r*_1_ values due to their larger size and the confinement of the Gd^3+^ ions in a sterically constrained space [8,9,10,11,12,13,14,15].

**Figure 5:**
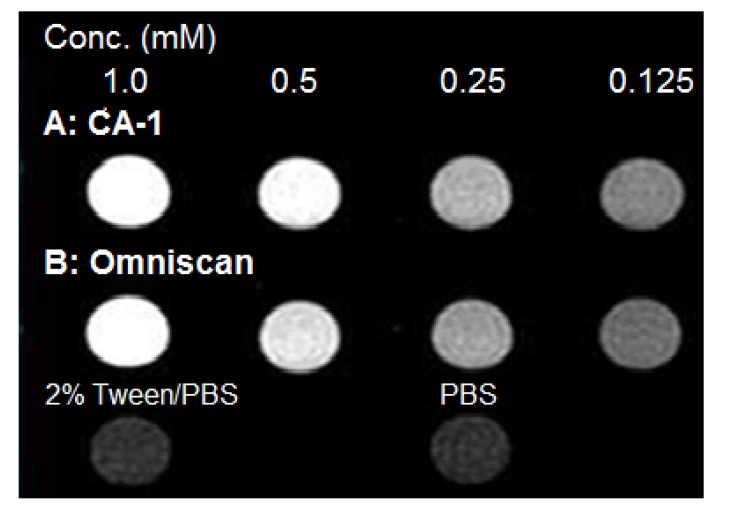
T1-weighted MRI images of **CA-1** and Omniscan at 7 T and 25 °C in PBS at different Gd^3+^ concentrations.

The T1-weighted MRI images of **CA-1** and Omniscan at 7 T at equimolar Gd^3+^ concentrations presented in Figure 5 shows greater contrast enhancement for **CA-1** compared to Omniscan due to its slightly higher relaxivity value. 

The possibility of aggregation in macromolecular CAs may affect their relaxivity values. To negate the possibility of aggregation of **CA-1** in solution, a dynamic light scattering (DLS) analysis of **CA-1** in PBS, 2% tween/PBS and bovine calf serum solution was performed. The average particle size of 11–13 nm was found in all formulations indicating no aggregation of the **CA-1** particles (SI). 

## 3. Experimental

### 3.1. General

Common reagents and chromatographic solvents were obtained from commercial suppliers (Sigma-Aldrich, Fisher Scientific) and used without any further purification. Lipophilic Sephadex LH-20 was obtained from GE Healthcare. DO3A-*t-*Bu-ester was purchased from Macrocyclics, Inc. NMR spectra were recorded on Bruker Avance 400 and 500 MHz spectrometers. The high-resolution mass spectrometry analysis was performed using Applied Biosystems Mariner ESI-TOF. IR spectra were recorded on Thermo Nicolet FT-IR spectrometer. The dynamic light scattering (DLS) analysis was performed on a Microtrac Zetatrac particle size analyzer. Gadolinium (Gd^3+^) concentrations of the samples used in MRI experiments were measured by inductively coupled plasma optical emission spectroscopy (ICP-OES) on a PerkinElmer OptimaTM 7,000 DV instrument. 

### 3.2. Synthesis

*Bis(tetrabutylammonium)-closo-dodecabromoacetoxydodecaborate* (**3**). A solution of TBA_2_-**2 **(0.50 g, 0.61 mmol) and bromoacetic anhydride (9.54 g, 36.71 mmol) in dry ACN (50 mL) was refluxed for 5 days in an argon atmosphere with vigorous stirring. Progress of the reaction was monitored by ^11^B NMR. Completion of the reaction was indicated by the appearance of a singlet at −17 ppm. The reaction mixture was then concentrated to dryness and purified using a size-exclusion column on Lipophilic Sephadex LH-20 with ACN as the eluent. The product was obtained as light brown semi-solid. Yield: 1.1 g (79%). IR (KBr): 3053, 2986, 2305, 1753, 1422, 1330, 1264 and 1231 cm^−1^. ^1^H-NMR (400 MHz, CDCl_3_): δ 3.92 (s, 24H), 3.12 (m, 16H), 1.62 (m, 16H), 1.42 (m, 16H), 0.99 (t, *J* = 7.2 Hz, 24H). ^13^C-NMR (100.6 MHz, CDCl_3_): δ 165.4, 59.8, 31.3, 24.7, 20.5 and 14.5. ^11^B-NMR (128 MHz, CDCl_3_): δ -17.70. HRMS (*m/z*): calcd. for C_24_H_24_B_12_Br_12_O_24_ [M]^2−^ 893.5956. Found: 893.5431 and calcd. for C_24_H_24_B_12_Br_12_O_24_+ C_16_H_36_N^1−^ (M+TBA) 2027.4798. Found: 2027.4041.

*Bis(tetrabutylammonium)-closo-dodecaazidoacetoxydodecaborate* (**4**): In a 100 mL round bottom flask, bis(tetrabutylammonium)-dodecabromoacetoxydodecaborate **3** (0.20 g, 0.09 mmol) and sodium azide (0.69 g, 10.57 mmol) were mixed with dry DMF (10 mL). This mixture was vigorously stirred at RT for 2 days under an argon atmosphere. The progress of the reaction was monitored by ^1^H-NMR. After completion, the reaction mixture was filtered through a celite pad and the filtrate was concentrated to dryness, the residue was re-dissolved in ethyl acetate and filtered again through a celite pad. The filtrate was collected and evaporated to dryness. The product was purified using size-exclusion column chromatography on Lipophilic Sephadex LH-20 with ACN as eluent. The product was obtained as a light brown semi-solid. Yield: 0.14 g (88%). All the spectroscopic characterization data was in perfect agreement with the reported data [19].

*3-(2-(2-(2-chloroethoxy)ethoxy)ethoxy)prop-1-yne* (**5**): To a two neck oven dried 250 mL round bottom flask, THF (50 mL) was added and the flask was placed in an ice bath. NaH (60% dispersion in oil, 2.8 g, 71.1 mmol) was added to it and the contents were stirred under argon atmosphere. 2-(2-(2-chloro)ethoxy)ethanol (10.0 g, 59.3 mmol) dissolved in THF (20 mL) was added to the reaction mixture over 15 minutes at 0 °C. The contents were stirred at 0 °C for 45 minutes and then propargyl bromide (10.5 g, 88.9 mmol) was added to it over a period of 30 minutes at 0 °C. The contents were then stirred at 0 °C for 2 h. After 2 h, reaction mixture was slowly heated to 60 °C and stirred at this temperature for 22 h. After 24 h, reaction mixture was allowed to cool to RT and an ice-cold aqueous solution of 5% HCl (100 mL) was added to it. The product was then extracted using ether (3 × 100 mL), the ether layer was washed with brine, dried over Na_2_SO_4_ and concentrated. This crude reaction mixture was purified using flash chromatography over silica gel to obtain product as very viscous pale yellow colored oil. Yield: 9.80 g (80%). ^1^H-NMR (400 MHz, CDCl_3_): δ 4.12 (d, *J* = 2.4 Hz, 2H), 3.69 (t, *J* = 6.0 Hz, 2H), 3.63–3.54 (m, 10H), 2.41 (t, *J* = 2.4 Hz, 1H). ^13^C-NMR (100.6 MHz, CDCl_3_): δ 80.4, 75.4, 72.1, 71.3, 71.3, 71.2, 69.8, 59.1, 43.5. HRMS (*m/z*): Calcd for C_9_H_15_ClO_3_ [M+Na]^+^ 229.0602. Found: 228.9594.

*3-(2-(2-(2-iodoethoxy)ethoxy)ethoxy)prop-1-yne* (**6**): A mixture of **5** (2.70 g, 13.0 mmol) and sodium iodide (3.91 g, 26.1 mmol) in acetone (30 mL) was stirred at 60 °C for 12 h. The reaction mixture was then cooled to RT and filtered. Filtrate was concentrated, and the crude material was purified on a silica gel column to obtain pure product as pale yellow oil. Yield: 3.7 g (95%). ^1^H-NMR (400 MHz, CDCl_3_): δ 4.16 (d, *J* = 2.4 Hz, 2H), 3.71 (t, *J* = 6.8 Hz, 2H), 3.66–3.61 (m, 8H), 3.21 (t, *J* = 7.2 Hz, 2H), 2.42 (t, *J* = 2.4 Hz, 1H). ^13^C-NMR (100.6 MHz, CDCl_3_): δ 80.5, 75.4, 72.7, 71.4, 71.2, 70.9, 69.9, 59.2, 43.5. HRMS (*m/z*): Calcd for C_9_H_15_IO_3_ [M+Na]^+^ 320.9958. Found: 320.9684.

*tri-tert-butyl2,2',2''-(10-(2-(2-(2-(prop-2-yn-1-yloxy)ethoxy)ethoxy)ethyl)-1,4,7,10 tetraazacyclododecane-1,4,7-triyl)triacetate* (**7**): To a mixture of DO3A-t-Bu-ester (2.00 g, 3.88 mmol) and **6** (1.73 g, 5.83 mmol) in DMF (25 mL), KHCO_3_ (0.78 g, 7.77 mmol) was added, and the mixture was stirred at 60 °C for 12 h. The reaction mixture was cooled and DCM (100 mL) added. The mixture was washed with brine (100 mL), and the organic layer was separated, dried over Na_2_SO_4_, and concentrated. The pure product was obtained as viscous oil after column chromatography over alumina (IV). Yield: 2.5 g (94%). ^1^H-NMR (400 MHz, CDCl_3_): δ 4.15 (d, J = 2.4 Hz, 2H), 3.65–3.57 (m, 10H), 3.53–3.16 (m, 2H), 3.14–2.96 (m, 4H), 2.92–2.67 (m, 7H), 2.66–2.53 (m, 4H), 2.50–2.38 (m, 4H), 2.37–2.18 (m, 4H), 1.45 (m, 27H). ^13^C-NMR (100.6 MHz, CDCl_3_): δ 172.8, 172.4, 82.1, 82.1, 79.5, 74.6, 70.1, 69.7, 69.6, 68.9, 67.0, 58.3, 56.3, 55.6, 52.1, 50.5, 49.7, 28.1, 27.9, 27.8. HRMS (m/z): Calcd for C_35_H_64_N_4_O_9_ Calcd for C_35_H_64_N_4_O_9_ [M+Na]^+^ 707.4566. Found: 707.5989.

*2,2',2''-(10-(2-(2-(2-(prop-2-yn-1-yloxy)ethoxy)ethoxy)ethyl)-1,4,7,10-tetraazacyclododecane-1,4,7-triyl)triacetic Acid* (**8**): A solution of **7** (0.25 g, 0.36 mmol) in formic acid (10 mL, 90%) was stirred for 12 h at 65 °C and then concentrated under reduced pressure. The residue was dissolved in a minimal amount of MeOH, and the product was precipitated by adding ether. Yield: 0.18 g (95%). Mp: 165 °C. IR (KBr): 3423, 2862, 1634, 1401, 1353, 1088, 693 cm^−1^. ^1^H-NMR (500 MHz, CDCl_3_): δ 5.16 (brs, 3H), 4.34 (m, 2H), 3.9 (m, 2H), 3.80–3.62 (m, 14H), 3.51–3.15 (m, 18H), 3.06 (t, *J* = 2.3 Hz, 1H). ^13^C-NMR (125.7 MHz, CDCl_3_): δ 172.5, 167.5, 100. 6, 82.6, 79.6, 77.5, 75.1, 70.1, 69.9, 69.9, 69.9, 69.9, 69.8, 69.8, 69.0, 68.9, 67.6, 57.7, 56.7, 55.6, 52.5, 50.7, 50.5, 49.9. HRMS (*m/z*): Calcd for C_23_H_40_N_4_O_9_ [M+H]^+^ 517.2874. Found: 517.3040 and Calcd for C_23_H_40_N_4_O_9_ [M+Na]^+^ 539.2693. Found: 539.3009.

*2,2',2''-(10-(2-(2-(2-(prop-2-yn-1-yloxy)ethoxy)ethoxy)ethyl)-1,4,7,10-tetraazacyclododecane-1,4,7-triyl)triacetic Acid Gd^3+^ Complex* (**9**): A mixture of **8** (0.21 g, 0.40 mmol) and Gd_2_O_3_ (0.09 g, 0.24 mmol) in H_2_O (10 mL) was stirred for 12 h at 100 °C. The solvent was removed under reduced pressure and the residue was dissolved in a minimum amount of methanol and passed through a celite pad to remove any solid present. Ether was added to precipitate an off-white powder which was then filtered, washed with ether and dried to obtain the product as a white solid. Yield: 0.25 g (91%). MP: 240 °C (decomposed). IR (KBr): 3440, 2865, 1612, 1443, 1383, 1323, 1086, 941, 723 cm^−1^. HRMS (m/z): Calcd for C_23_H_37_GdN_4_O_9_ [M+Na]^+^ 694.1700. Found: 694.1610 and Calcd for C_23_H_37_GdN_4_O_9_ [3M+2Na]^2+^ 1029.7600. Found: 1029.7294 and C_23_H_37_GdN_4_O_9_ [2M+Na]^+^ 1364.3509. Found: 1364.4569.

*2,2',2''-(10-(2-(2-(2-(prop-2-yn-1-yloxy)ethoxy)ethoxy)ethyl)-1,4,7,10-tetraazacyclododecane-1,4,7-triyl)triacetic Acid Dy^3+^ Complex* (**10**): A mixture of **8** (0.37 g, 0.72 mmol) and DyCl_3_·6H_2_O (0.27 g, 0.72 mmol) in pyridine (10 mL) was stirred for 12 h at 70 °C. The solvent was removed under reduced pressure and the residue was dissolved in a minimum amount of methanol and passed through a celite pad to remove any solid present. Ether was added to precipitate an off-white powder which was then filtered, washed with ether and dried to obtain the product as a white solid. Yield: 0.45 g (92%). MP: 180 °C (decomposed). IR (KBr): 3425, 2869, 1611, 1444, 1383, 1321, 1087, 940, 723 cm^−1^. HRMS (m/z): Calcd for C_23_H_37_DyN_4_O_9_ [M+Na-H]^+^ 699.1672. Found: 699.0800 and Calcd for C_23_H_37_DyN_4_O_9_ [M+Na+Li-H]^+^ 706.1832. Found: 706.3500.

*Synthesis of Closomer* **11**: A mixture of 12-fold azidoacetate closomer **4 (**0.07 g, 0.04 mmol) and DO3A ligand **7** (1.26 g, 1.85 mmol) was dissolved in ACN-THF (20 mL, 50:50 mixture). To this mixture, DIPEA (0.60 g, 4.62 mmol) and copper (I) iodide (0.09 g, 0.46 mmol) were added and the resulting mixture was stirred for 5 days at RT under an argon atmosphere. The reaction mixture was then concentrated to dryness and the residue was dissolved in DCM and filtered. The filtrate was washed with an aqueous 2% ethylenediaminetetraacetic acid disodium salt (EDTA.2Na) solution to remove the trace of copper ions and the organic layer was separated, dried and concentrated. The closomer **11** was purified via size-exclusion chromatography over Lipophilic Sephadex LH-20 using ACN as eluent. Yield: 0.26 g (67%), obtained as viscous oil. IR (neat): 3441, 2975, 2933, 2868, 1727, 1457, 1386, 1310, 1227, 1161, 1112, 848, 732 cm^−1^. ^1^H-NMR (400 MHz, CD_3_CN): δ 7.74 (s, 12H), 4.96 (s, 24H), 4.58 (m, 24H), 3.84 9m, 24H), 3.59–3.26 (m, 200H), 3.03–2.98 (m, 52H), 2.80–2.29 (m, 172H), 1.46 (m, 331H), extra proton counts are due to the presence of H_3_O^+^ and TBA^+^ cations. ^13^C-NMR (100.6 MHz, CD_3_CN): δ 173.8, 173.6, 171.3, 171.1, 166.4, 145.1, 125.9, 82.7, 81.9, 81.8, 73.2, 71.0, 70.9, 70.7, 70.6, 70.3, 70.2, 70.0, 69.8, 68.1, 65.9, 64.6, 61.5, 58.7, 57.2, 57.0, 56.3, 55.7, 55.6, 53.7, 53.3, 53.1, 52.8, 51.2, 50.7, 48.4, 28.4, 28.3, extra carbon counts are due to the presence of TBA^+^ cations. ^11^B-NMR (128 MHz, CD_3_CN): δ −17.90. HRMS (*m/z*): Average mass calcd for C_444_H_792_B_12_N_84_O_132_ [M] 9549.2549. Found: 1689 [M^6+^+4Na+4H], 1483 [M^7+^+5Na+4H], 1247 [M^8+^+2Na+8H], 1159 [M^9+^+ 4Na+7H].

#### *Synthesis of* **CA-1**

**Route A**. Closomer **11** (0.20 g, 0.02 mmol) was treated with 80% TFA/DCM (10 mL) for 6 h at RT under an argon atmosphere. The reaction mixture was concentrated under reduced pressure and the resulting residue was dissolved in a minimum amount of MeOH and precipitated by adding ether. The solid was filtered, washed with ether and dried under high vacuum overnight. The solid was then dissolved in 1M citrate buffer (15 mL, pH-7) and added slowly to a solution of GdCl_3_.6H_2_O (0.74 g, 1.19 mmol) in 15 mL of 1M citrate buffer over 5 h at RT with vigorous stirring. The pH of the reaction mixture was maintained at approximately 7 using 0.3 N NaOH. The reaction mixture was stirred for additional 15 h at RT and was sonicated a few times during the course of the reaction. The reaction mixture was then dialyzed in deionized water for 2 days using 1,000 MWCO membrane tubes (Spectra/Por^®^). The product **CA-1** was obtained as an off-white solid after lyophilization. Yield: 0.14 g (75%). MP: 260 °C (decomposed). IR (KBr): 3425, 2871, 1744, 1601, 1383, 1324, 1260, 1086, 718 cm^−1^. ICP-OES analysis: calcd average Gd per closomer: 12. Found: 12. HRMS-ESI-TOF (*m/z*): Calcd for B_12_C_300_H_468_Gd_12_N_84_O_132_ [M]^2−^ 4692.2286. Found: 4692.8213.

**Route B**. Pre-complexation. A mixture of 12-fold azidoacetate closomer **4 (**0.05 g, 0.03 mmol), CuSO_4_.5H_2_O (12.4 mg, 0.05 mmol), sodium ascorbate (20.0 mg, 0.10 mmol) and DO3A Gd^3+^ complex **9** (0.66 g, 1.00 mmol) was dissolved in ACN-H_2_O (30 mL, 50:50 mixture). This mixture was stirred for 3 days at 60 °C. The reaction mixture was then cooled, filtered and the filtrate was concentrated to dryness and the residue was purified via size-exclusion chromatography over Lipophilic Sephadex LH-20 using water as eluent. The product **CA-1** was obtained as an off-white solid after lyophilization. Yield: 0.10 g (38%). 

### 3.3. Relaxivity Determinations

Solutions of **CA-1** and Omniscan were prepared in PBS, 2% Tween-80/PBS and bovine calf serum. T1 relaxation rates were determined for each of the three formulations of **CA-1** at Gd^3+^ ion concentrations of 0.125, 0.25, 0.5 and 1 mM. The Gd^3+^ ion concentration of Omniscan was 1 mM. The measurements were repeated on two or more independently prepared samples to ensure consistency. A buffer matched blank sample (0 mM) was also used in the relaxivity measurements of each sample. 

Measurements were performed using a 7 Tesla Varian Unity Inova MRI system (Varian Inc./Agilent Technologies) at 25 °C. A T1-weighted MRI pulse sequence was applied with TE = 15 ms, and TR = 500 ms, slice thickness = 1 mm, matrix = 256 × 256, and FOV = 30 × 30 mm. A series of inversion recovery (IR) spin-echo images were acquired using TR = 3 s, TE = 15 ms, and the following inversion delays: 0.082, 0.1, 0.12, 0.16, 0.24, 0.32, 0.64, 1.28, 2.56, 3.6 s. Water signal intensities were measured using VnmrJ2.1D software (Varian Inc./Agilent Technologies, 2005). Relaxation rates were calculated using a three-parameter exponential recovery fitting in Origin 8.5.0 (OriginLab Corporation, 2012). The final relaxivity values were obtained by linear fitting of relaxation rates against concentrations for each sample.

## 4. Conclusions

In summary, we report the synthesis of a novel multimeric MRI contrast agent (**CA-1**) covalently attached to a icosahedral *closo*-borane core via Cu(I) catalyzed azide-alkyne click chemistry approach. This unique macromolecular CA is highly water soluble and the preliminary relaxivity studies confirms that **CA-1** exhibits higher relaxivity values (per Gd relaxivity: *r*_1_ = 4.7 mM^−1^s^−1^, and per molecule *r*_1_: = 56.5 mM^−1^s^−1^ at 7 T and 25 °C in PBS) as compared to the frequently used clinical MRI CAs. The detailed stability, toxicity and *in vivo* MRI assessments of **CA-1** is currently in progress. We also investigated a more efficient route for the synthesis of the 12-fold azidoacetate closomer **4** from *closo*-B_12_(OH)_12_^2−^ through the intermediary of 12-fold bromoacetate closomer **3**. The 12-fold azidoacetate closomer **4** is thus demonstrated to be a vital scaffold for the development of a wide variety of high-payload, receptor-specific diagnostic and therapeutic agents via click chemistry approach.

## Supplementary Materials

Supplementary File 1
