# Supplementary material for: Synthesis and Relaxivity Studies of a DOTA-Based Nanomolecular Chelator Assembly Supported by an Icosahedral Closo-B122− -Core for MRI: A Click Chemistry Approach
Source: Molecules. 2013 Jul 29;18(8):9034–48. doi: 10.3390/molecules18089034 (PMC6270972; doi:10.3390/molecules18089034)

Synthesis and Relaxivity Studies of a DOTA-based Nanomolecular Chelator Assembly Supported by an Icosahedral *Closo*-B_{12}^{2-} -Core for MRI: A Click Chemistry Approach

Lalit N. Goswami, Lixin Ma, Peter J. Kueffer, Satish S. Jalisatgi and M. Frederick Hawthorne*
International Institute of Nano and Molecular Medicine, School of Medicine, University of Missouri, Columbia, Missouri 65211-3450
*Author to whom correspondence should be address; Email: hawthornem@missouri.edu;
Tel.: 001-573-882-7016; Fax: 001-573-884-6900.

Supporting Information (SI)

^{1}H, ^{13}C, ^{11}B NMR, HRMS Spectra	**2-26**
IR spectra of closomer **4**, **11** and **CA-1**	**27-29**
Determination of hydration number (q)	**30**
Comparison of relaxivity	**31**
Dynamic Light Scattering Analysis of **CA-1**	**32**
HPLC analysis of **CA-1**	**33**

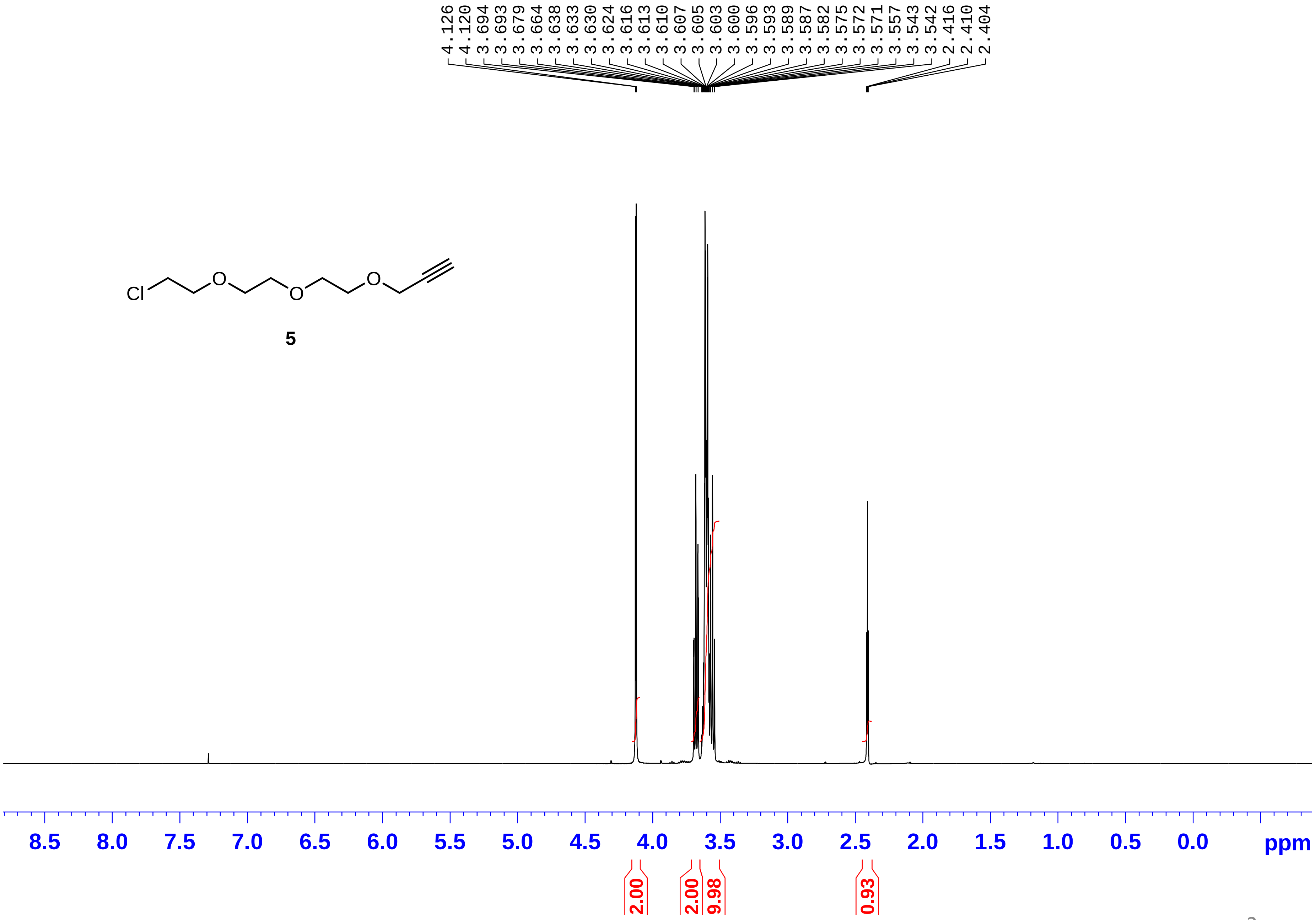
4.126
4.120
3.694
3.693
3.679
3.664
3.638
3.633
3.630
3.624
3.616
3.613
3.610
3.607
3.605
3.603
3.600
3.596
3.593
3.589
3.587
3.582
3.575
3.572
3.571
3.557
3.543
3.542
2.416
2.410
2.404
Cl
O
O
O
5
8.5
8.0
7.5
7.0
6.5
6.0
5.5
5.0
4.5
4.0
3.5
3.0
2.5
2.0
1.5
1.0
0.5
0.0
ppm
2.00
2.00
9.98
0.93

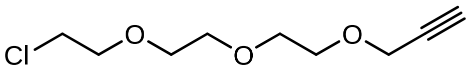
Cl
O
O
O

5

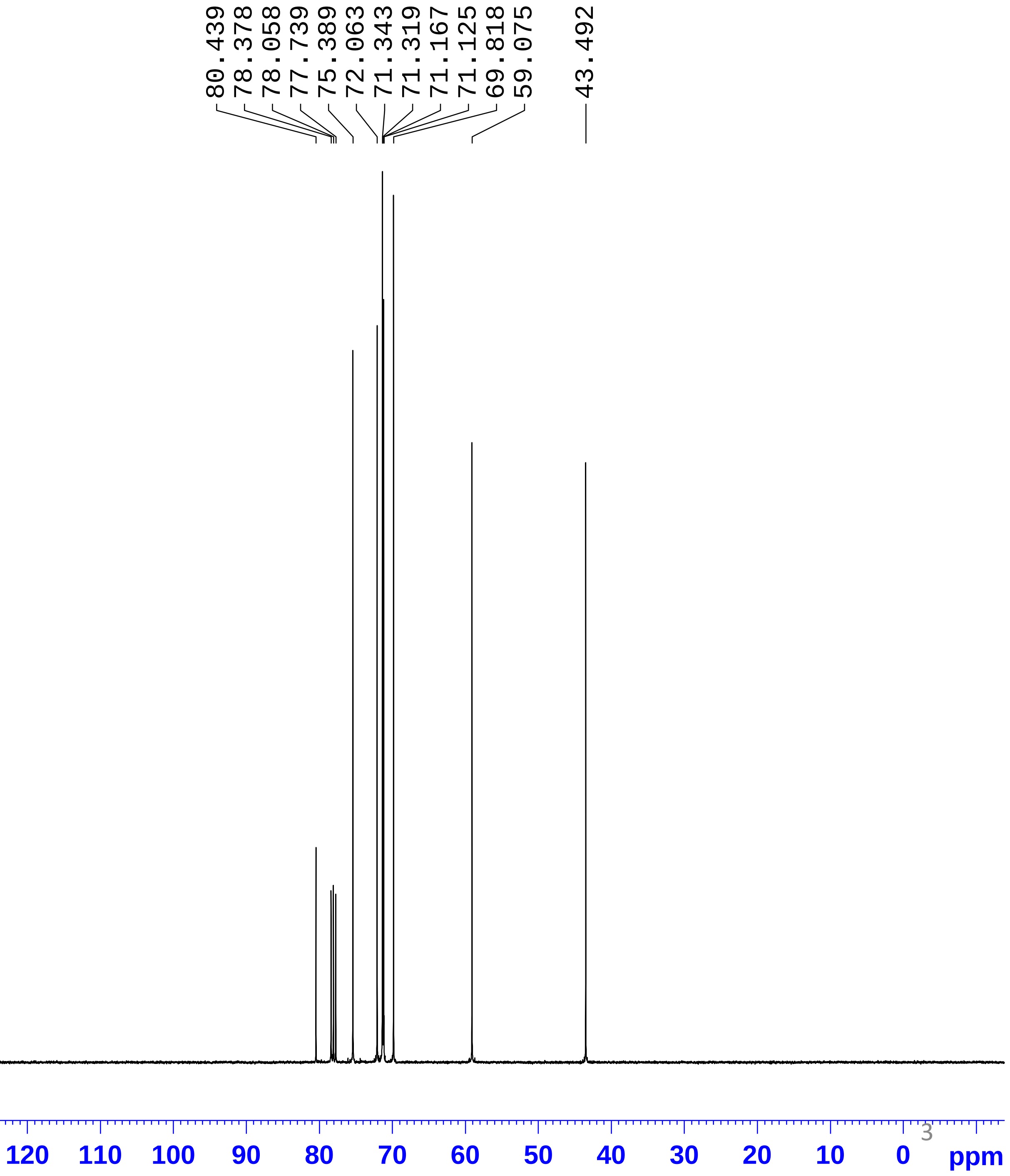
80.439
78.378
78.058
77.739
75.389
72.063
71.343
71.319
71.167
71.125
69.818
59.075
43.492
190 180 170 160 150 140 130 120 110 100 90 80 70 60 50 40 30 20 10 0 ppm

Applied Biosystems Mariner System 5268

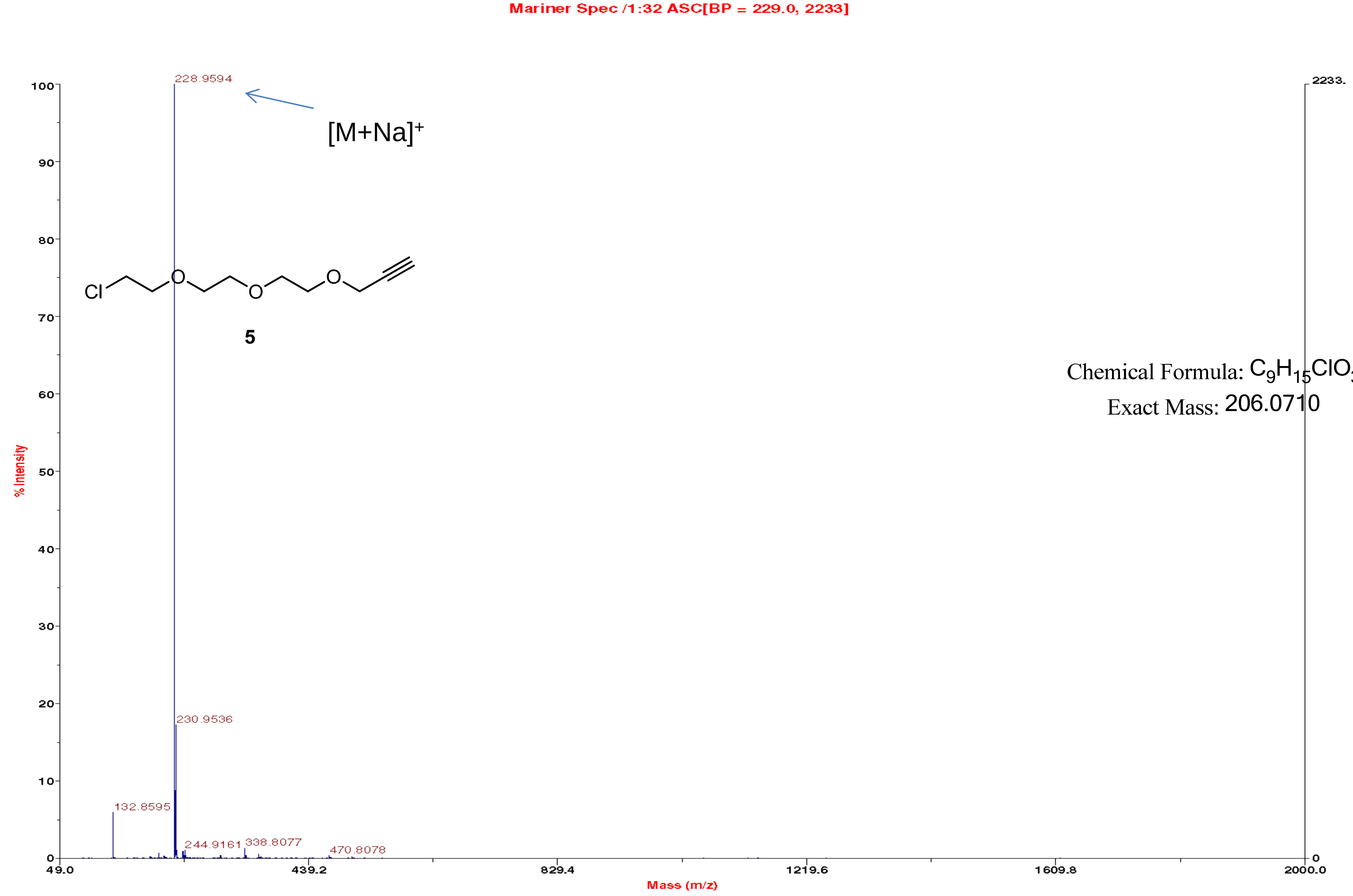

Chemical Formula: $C_9H_{15}ClO_3$

Exact Mass: 206.0710

Acquired: Apr 01 15:06:00 2010
Mariner Mass Spectrum
C:\Mariner\Data\2010\Apr\01 Thur\LNG-508M003.dat

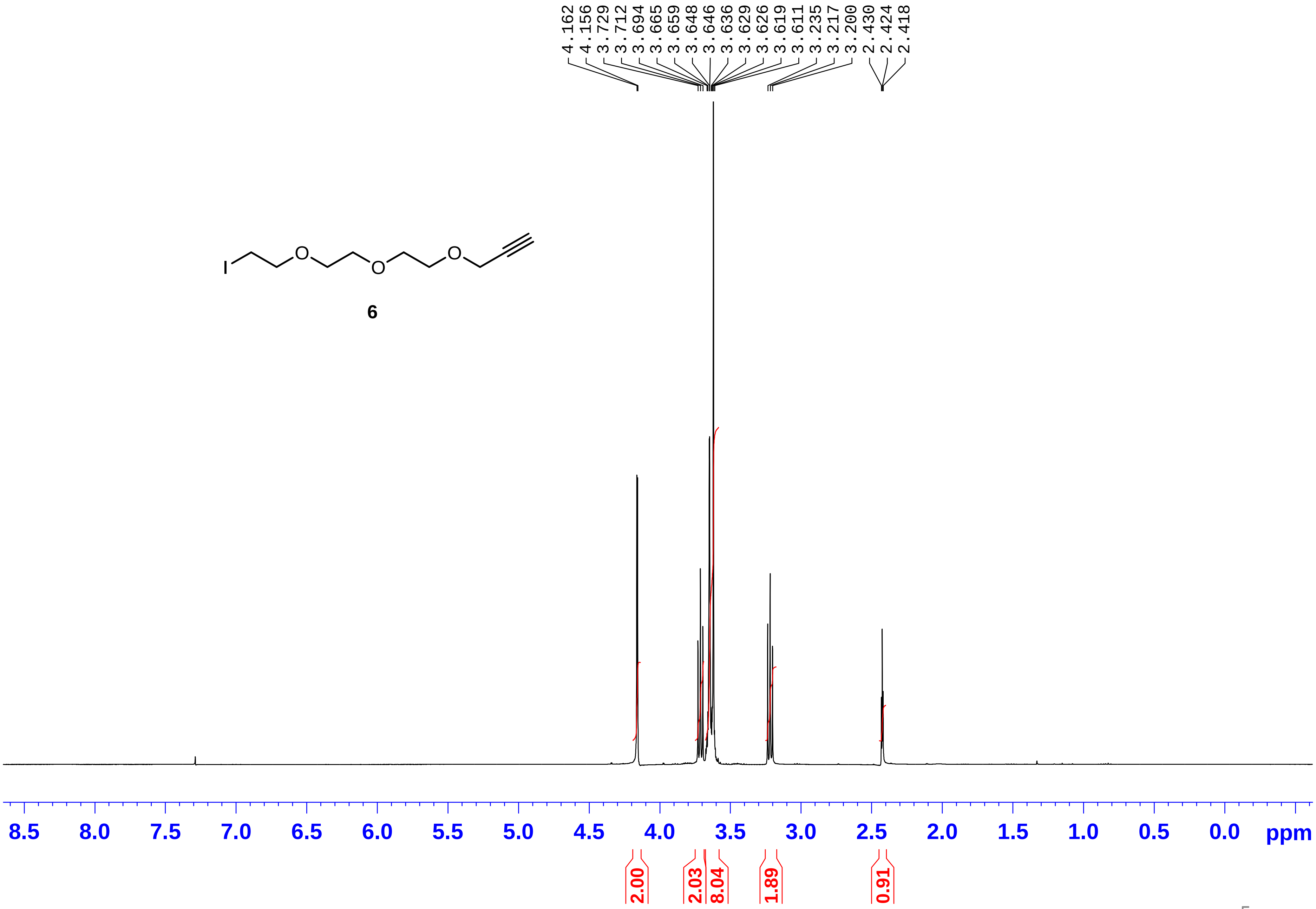
4.162
4.156
3.729
3.712
3.694
3.665
3.659
3.648
3.646
3.636
3.629
3.626
3.619
3.611
3.235
3.217
3.200
2.430
2.424
2.418
I
O
O
O
6
8.5
8.0
7.5
7.0
6.5
6.0
5.5
5.0
4.5
4.0
3.5
3.0
2.5
2.0
1.5
1.0
0.5
0.0
ppm
2.00
2.03
8.04
1.89
0.91

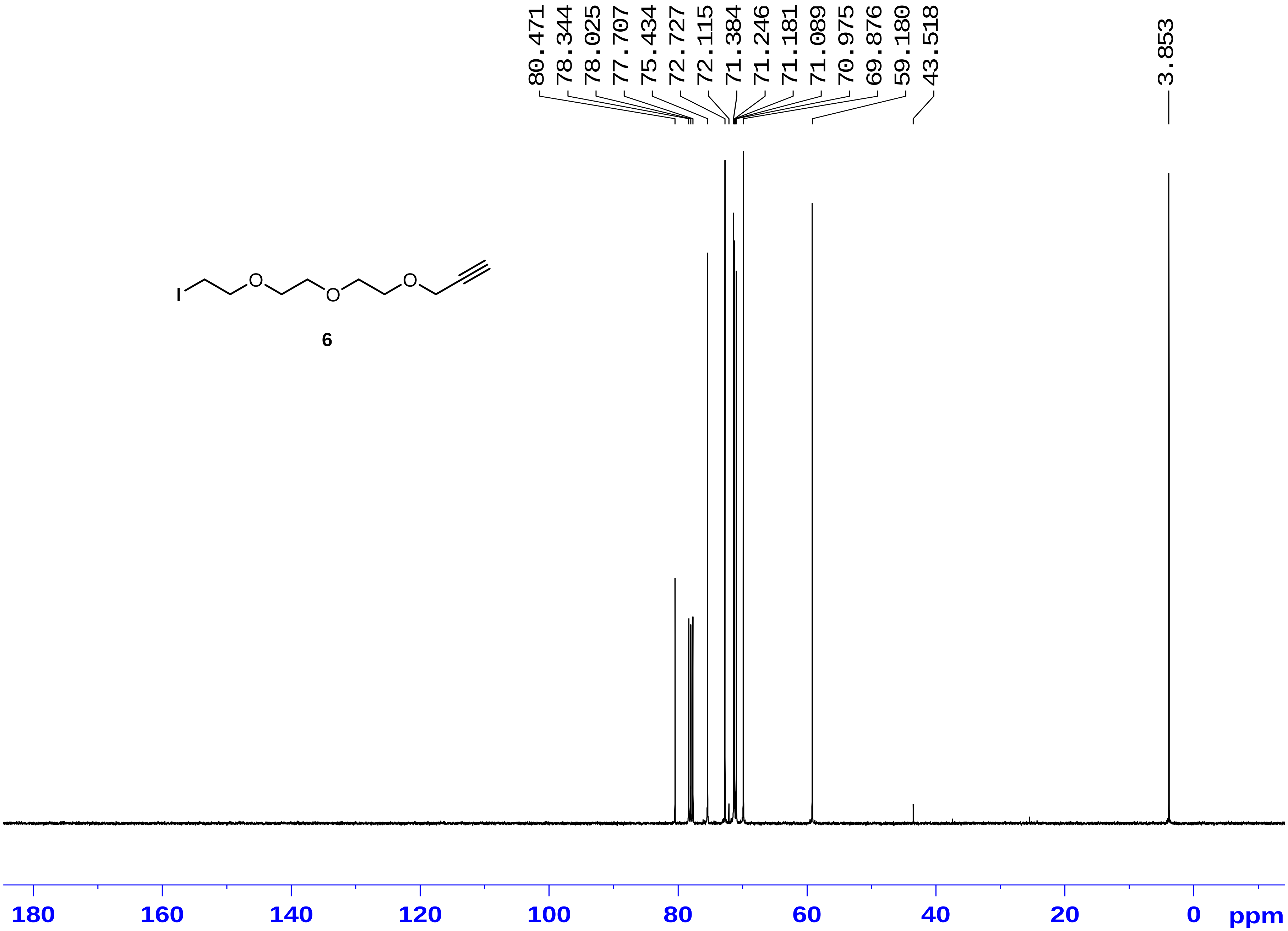
I
O
O
O
6
80.471
78.344
78.025
77.707
75.434
72.727
72.115
71.384
71.246
71.181
71.089
70.975
69.876
59.180
43.518
3.853
180
160
140
120
100
80
60
40
20
0
ppm

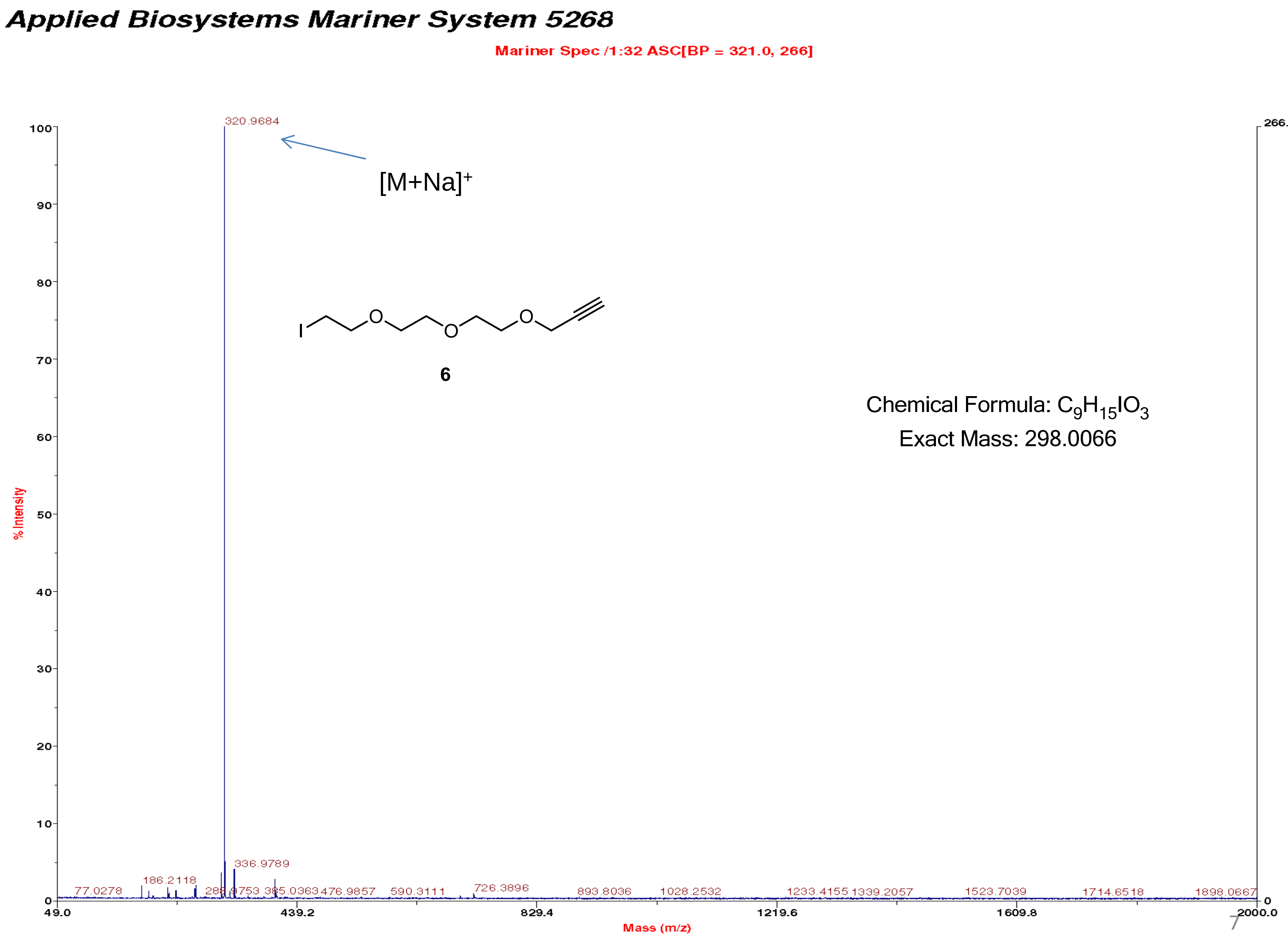

Applied Biosystems Mariner System 5268
Mariner Spec /1:32 ASC[BP = 321.0, 266]
320.9684
[M+Na]+
336.9789
186.2118
77.0278
726.3896
590.3111
476.9857
893.8036
1028.2532
1233.4155
1339.2057
1523.7039
1714.6518
1898.0667
266.4
% Intensity
Mass (m/z)
49.0
439.2
829.4
1219.6
1609.8
2000.0
6
Chemical Formula: C9H15IO3
Exact Mass: 298.0066

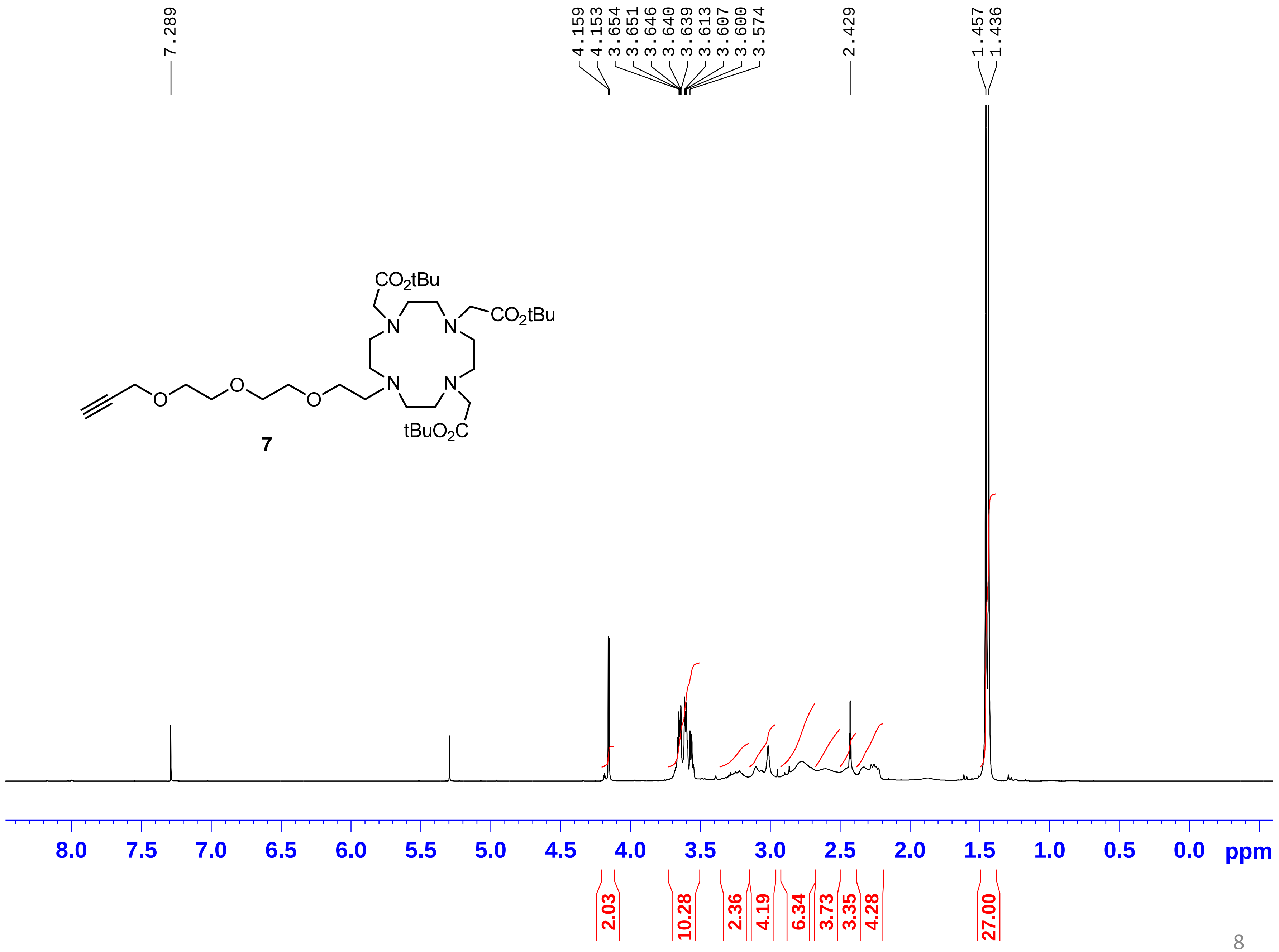
7.289
4.159
4.153
3.654
3.651
3.646
3.640
3.639
3.613
3.607
3.600
3.574
2.429
1.457
1.436
CO2tBu
CO2tBu
tBuO2C
N
O
7
8.0 7.5 7.0 6.5 6.0 5.5 5.0 4.5 4.0 3.5 3.0 2.5 2.0 1.5 1.0 0.5 0.0 ppm
2.03
10.28
2.36
4.19
6.34
3.73
3.35
4.28
27.00

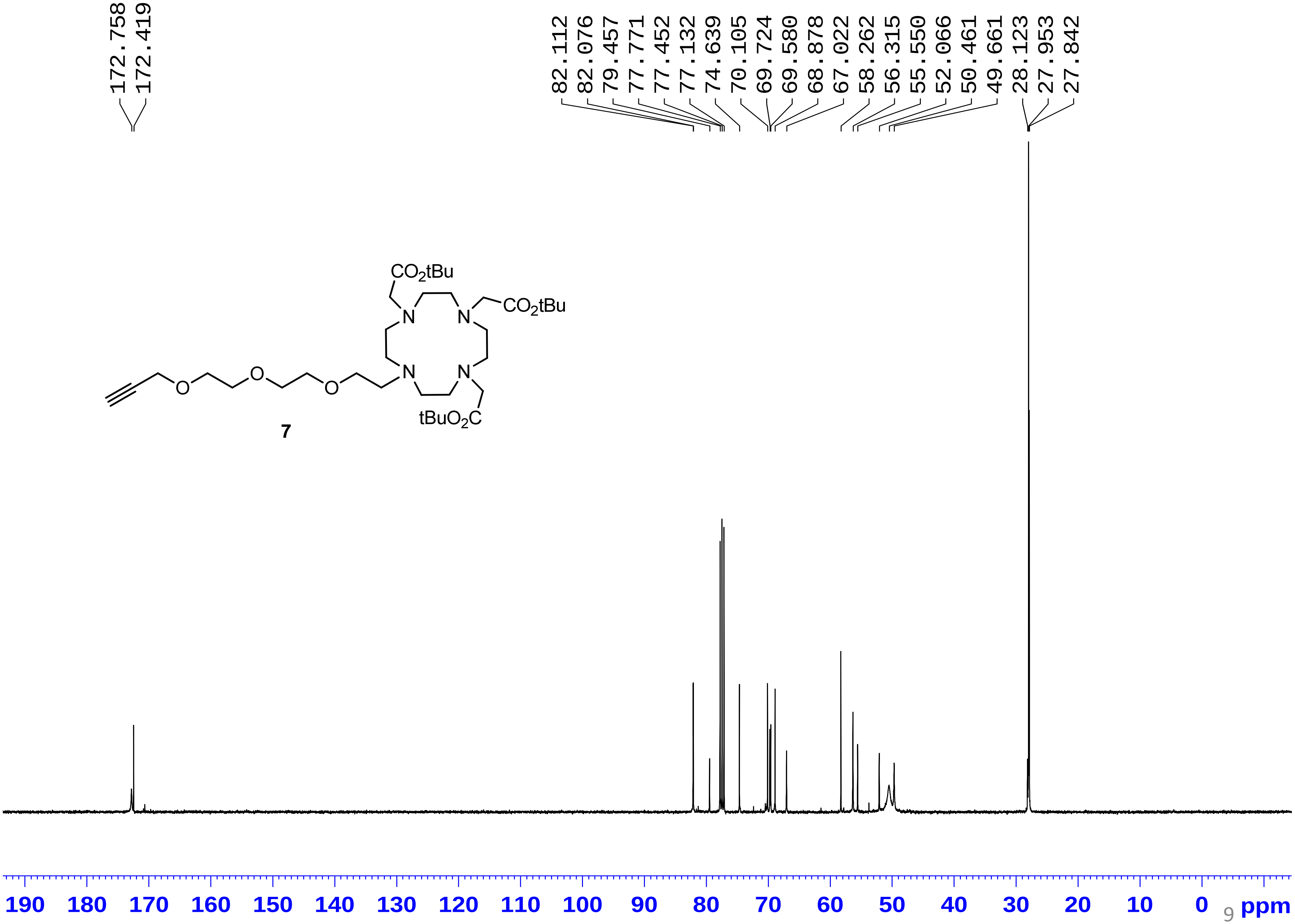
172.758
172.419
82.112
82.076
79.457
77.771
77.452
77.132
74.639
70.105
69.724
69.580
68.878
67.022
58.262
56.315
55.550
52.066
50.461
49.661
28.123
27.953
27.842
CO2tBu
CO2tBu
tBuO2C
N
O
7
190 180 170 160 150 140 130 120 110 100 90 80 70 60 50 40 30 20 10 0 ppm

Applied Biosystems Mariner System 5268

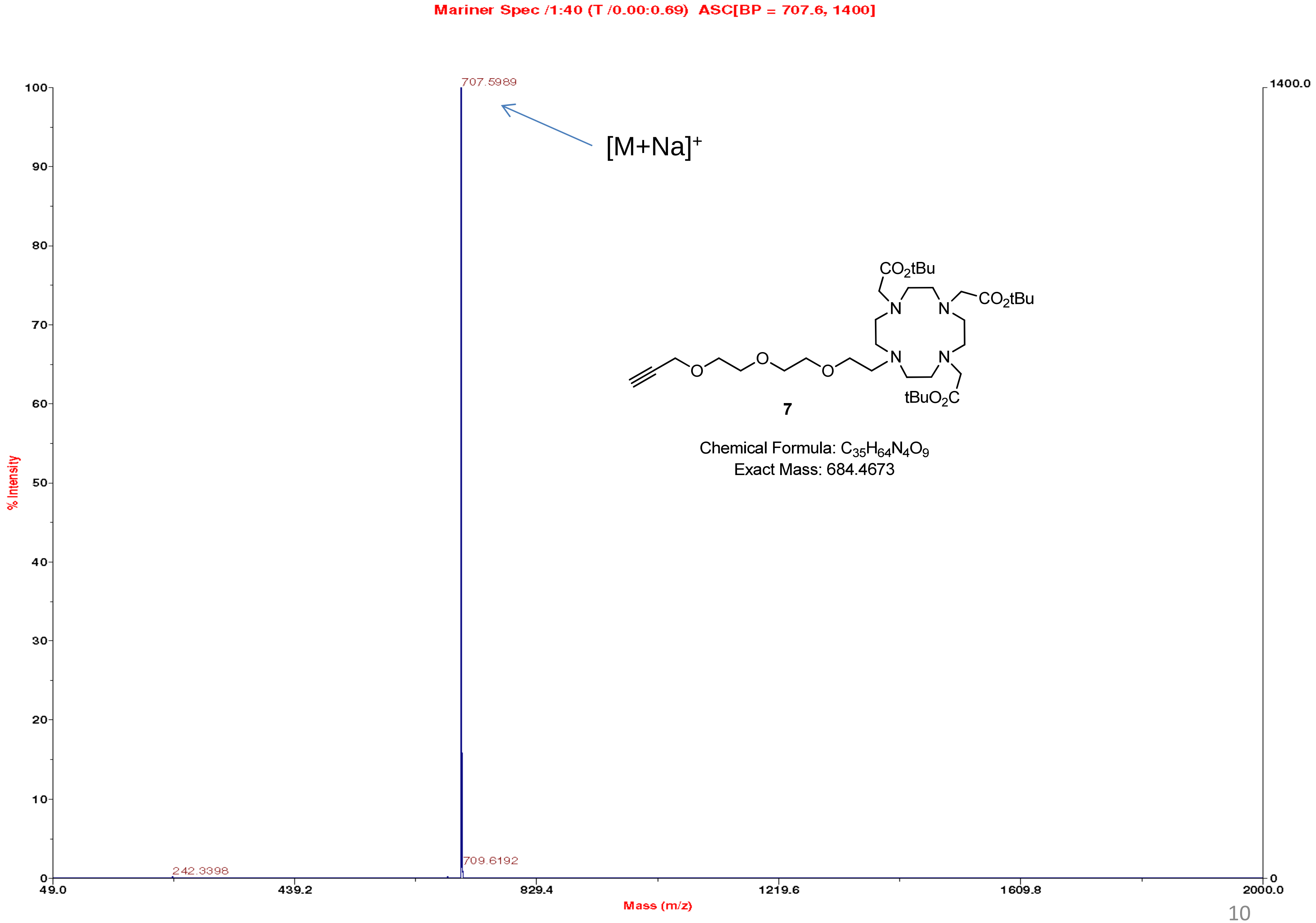

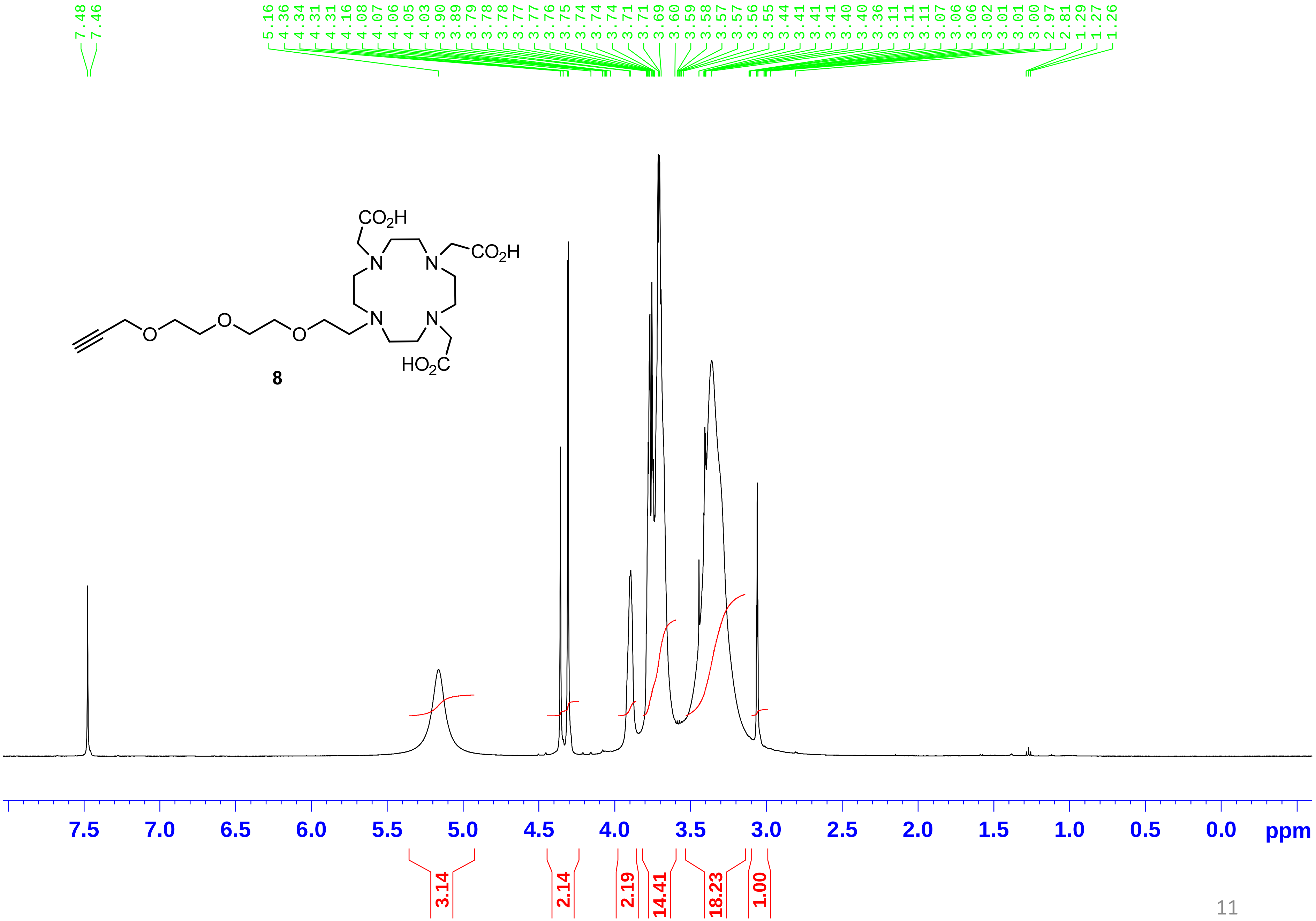

7.48
7.46
5.16
4.36
4.34
4.31
4.31
4.16
4.08
4.07
4.06
4.05
4.03
3.90
3.89
3.79
3.78
3.78
3.77
3.76
3.75
3.74
3.74
3.74
3.71
3.71
3.69
3.60
3.59
3.58
3.57
3.57
3.56
3.55
3.44
3.41
3.41
3.40
3.40
3.36
3.11
3.11
3.07
3.06
3.06
3.02
3.01
3.01
3.00
2.97
2.81
1.29
1.27
1.26
CO2H
CO2H
HO2C
N
N
N
N
O
O
O
8
7.5
7.0
6.5
6.0
5.5
5.0
4.5
4.0
3.5
3.0
2.5
2.0
1.5
1.0
0.5
0.0
ppm
3.14
2.14
2.19
14.41
18.23
1.00

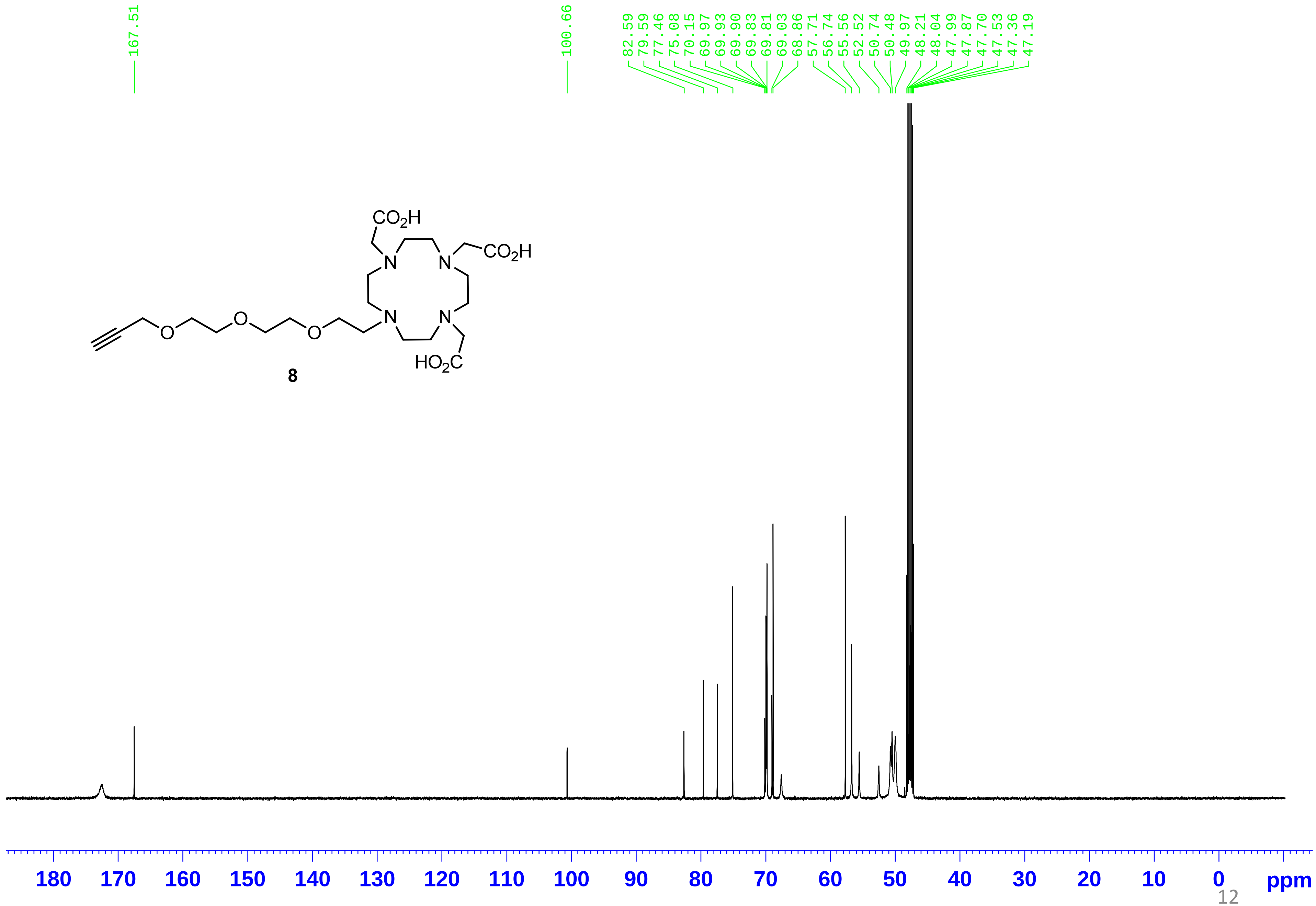
167.51
100.66
82.59
79.59
77.46
75.08
70.15
69.97
69.93
69.90
69.83
69.81
69.03
68.86
57.71
56.74
55.56
52.52
50.74
50.48
49.97
48.21
48.04
47.99
47.87
47.70
47.53
47.36
47.19
CO2H
CO2H
HO2C
N
O
8
180 170 160 150 140 130 120 110 100 90 80 70 60 50 40 30 20 10 0 ppm

Applied Biosystems Mariner System 5268

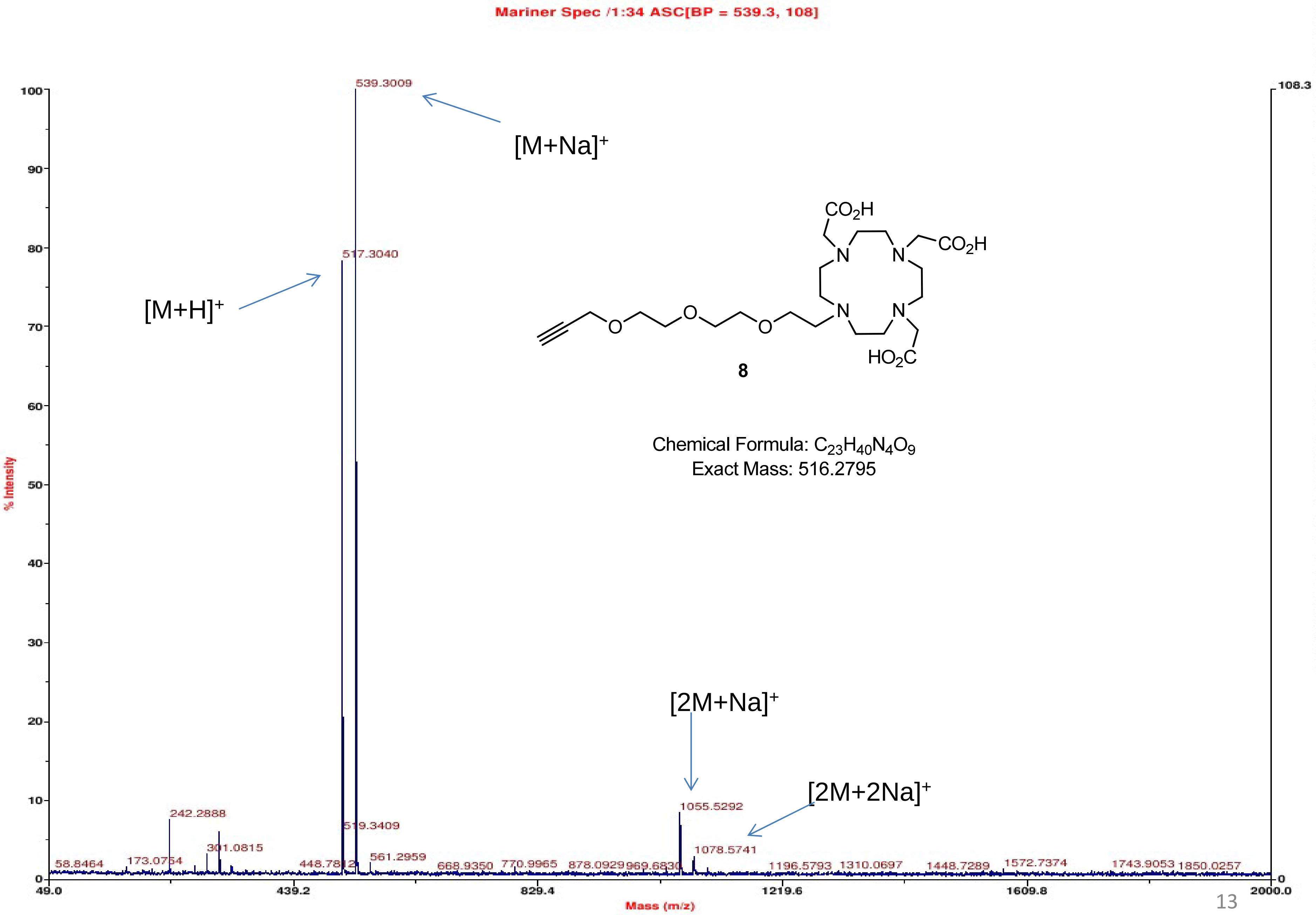

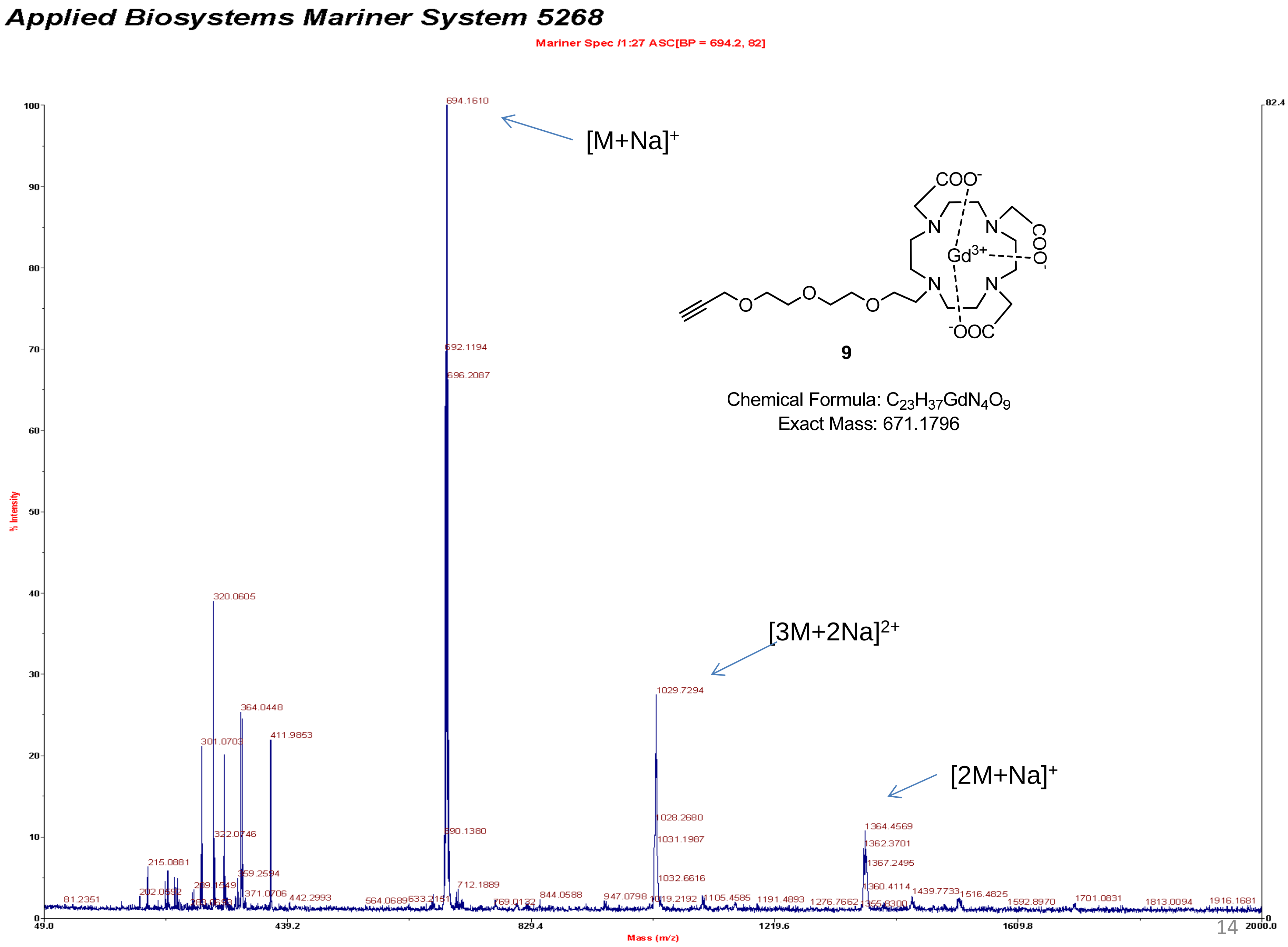
Applied Biosystems Mariner System 5268
Mariner Spec /1:27 ASC[BP = 694.2, 82]
% Intensity
Mass (m/z)
694.1610
[M+Na]+
692.1194
696.2087
320.0605
364.0448
411.9853
301.0703
215.0881
322.0746
1029.7294
[3M+2Na]2+
1028.2680
1031.1987
1032.6616
[2M+Na]+
1364.4569
1362.3701
1367.2495
1360.4114
1439.7733
1516.4825
9
Chemical Formula: C23H37GdN4O9
Exact Mass: 671.1796

Applied Biosystems Mariner System 5219

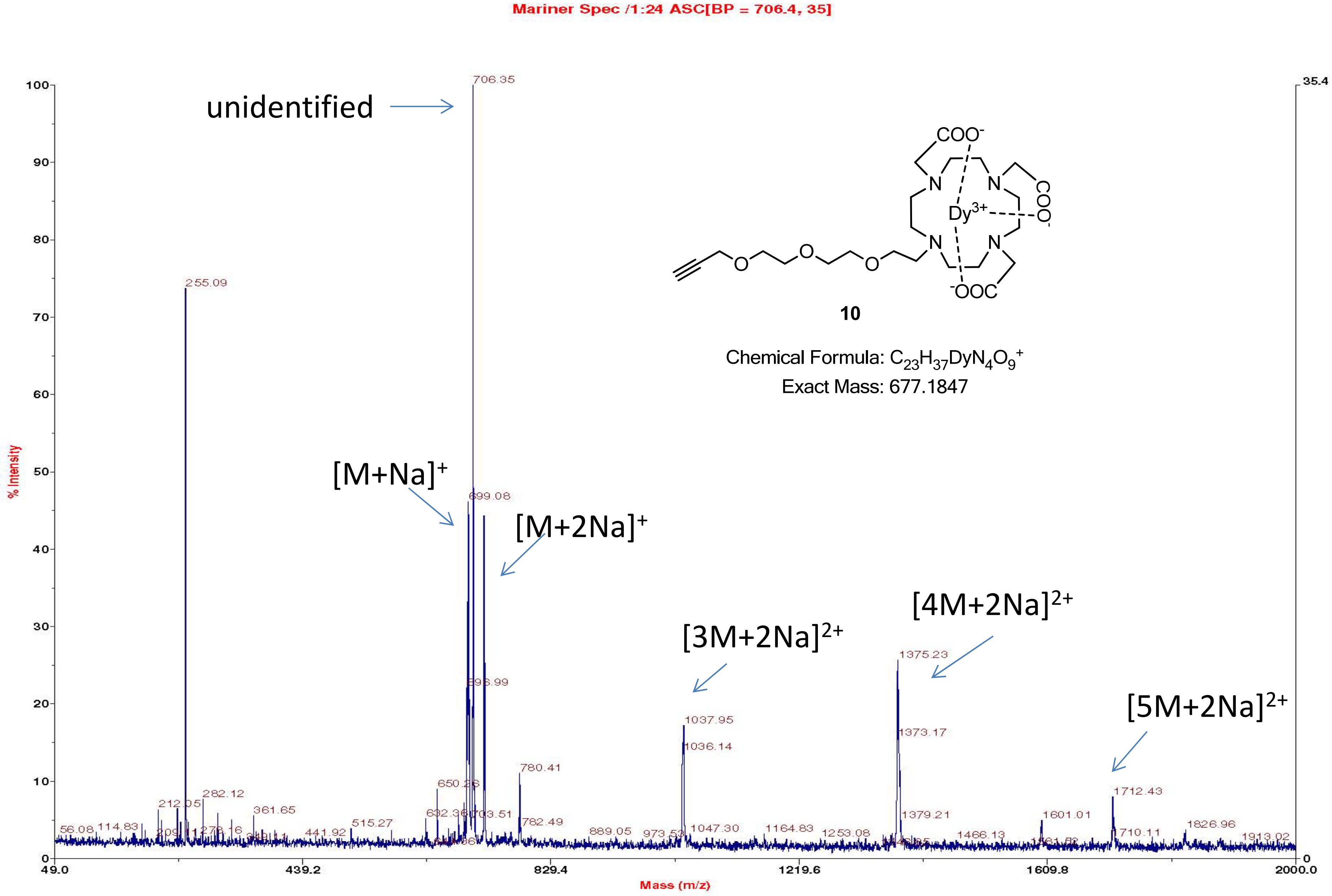

Acquired: Sep 04 16:50:00 2012
Mariner Mass Spectrum
C:\Mariner\Data\2012\Sep\04 Tue\LNG-1018C002.dat

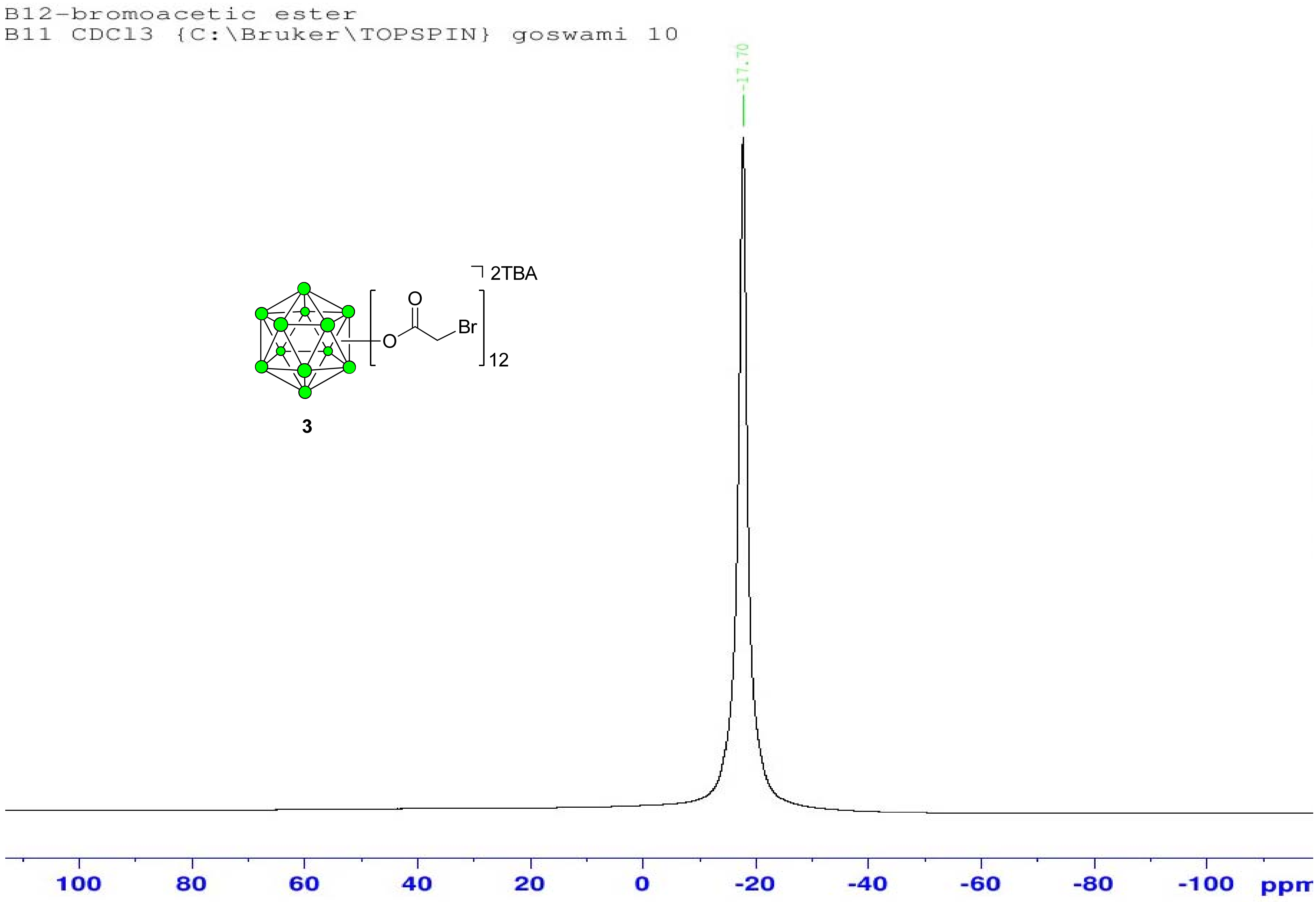
B12-bromoacetic ester
B11 CDCl3 {C:\Bruker\TOPSPIN} goswami 10
-17.70
2TBA
O
O
Br
12
3
100
80
60
40
20
0
-20
-40
-60
-80
-100
ppm

B12-bromoacetic ester
proton CDCl3 {C:\Bruker\TOPSPIN} goswami 9

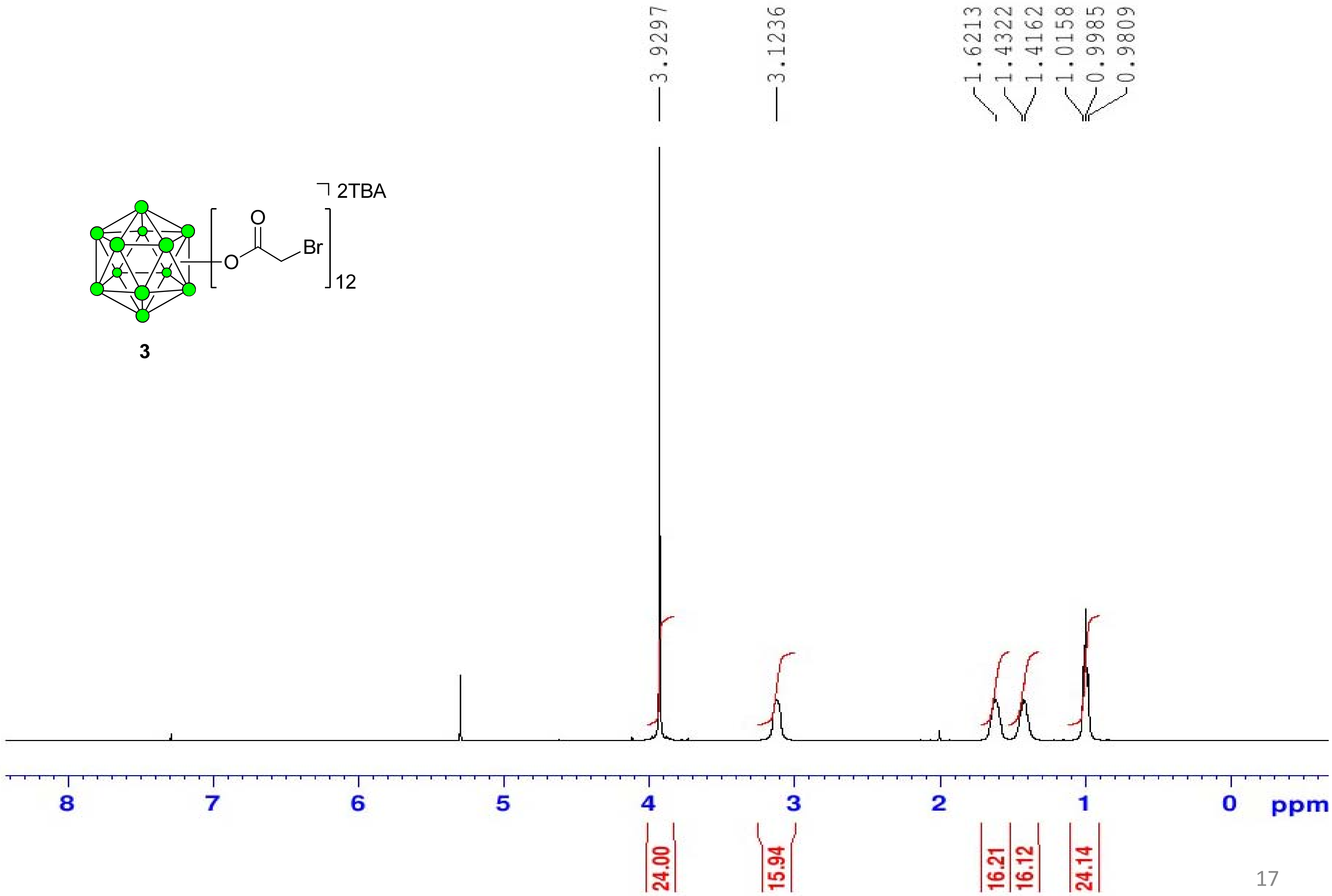

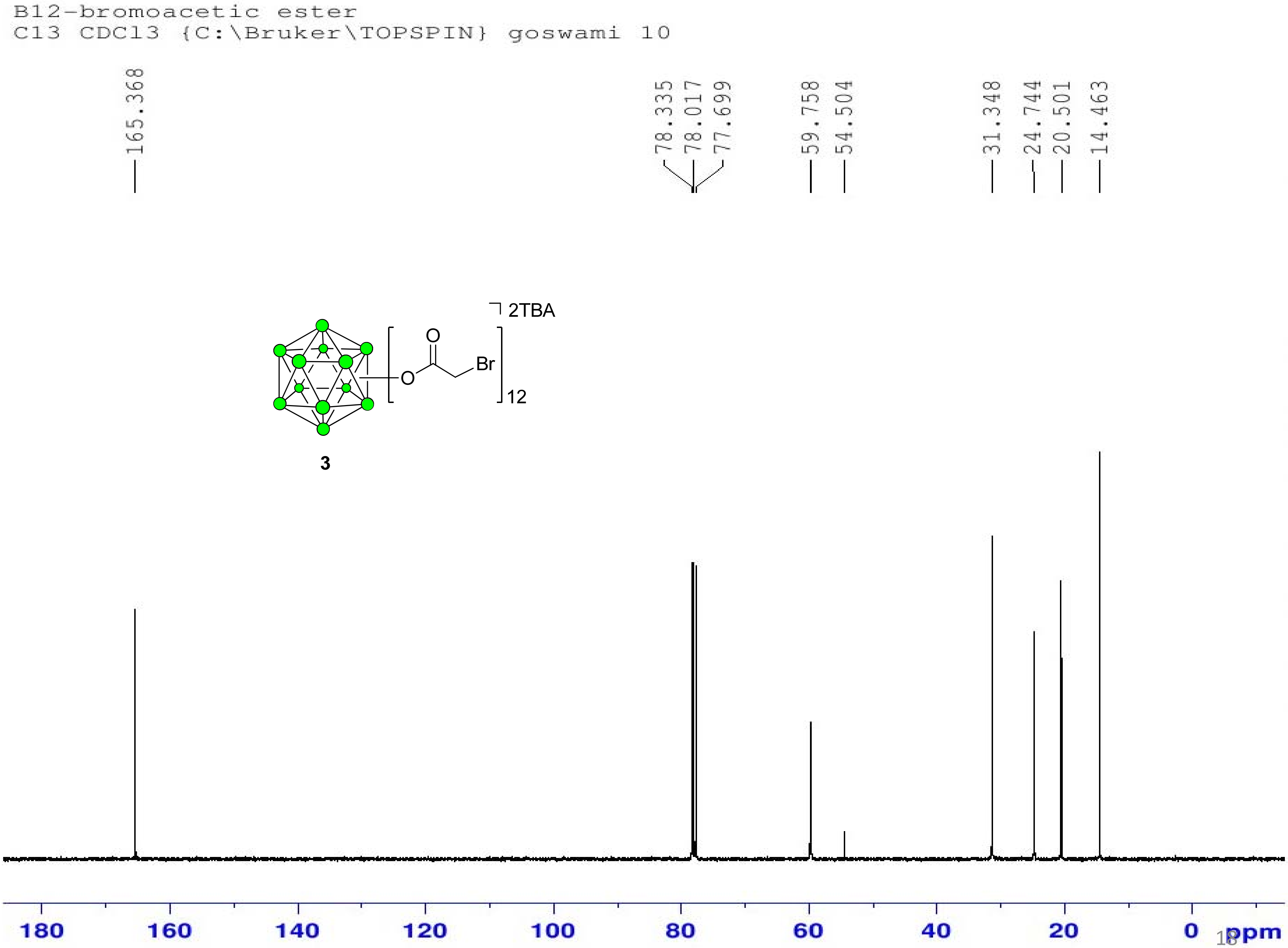
B12-bromoacetic ester
C13 CDCl3 {C:\Bruker\TOPSPIN} goswami 10
165.368
78.335
78.017
77.699
59.758
54.504
31.348
24.744
20.501
14.463
2TBA
O
O
Br
12
3
180 160 140 120 100 80 60 40 20 0 ppm

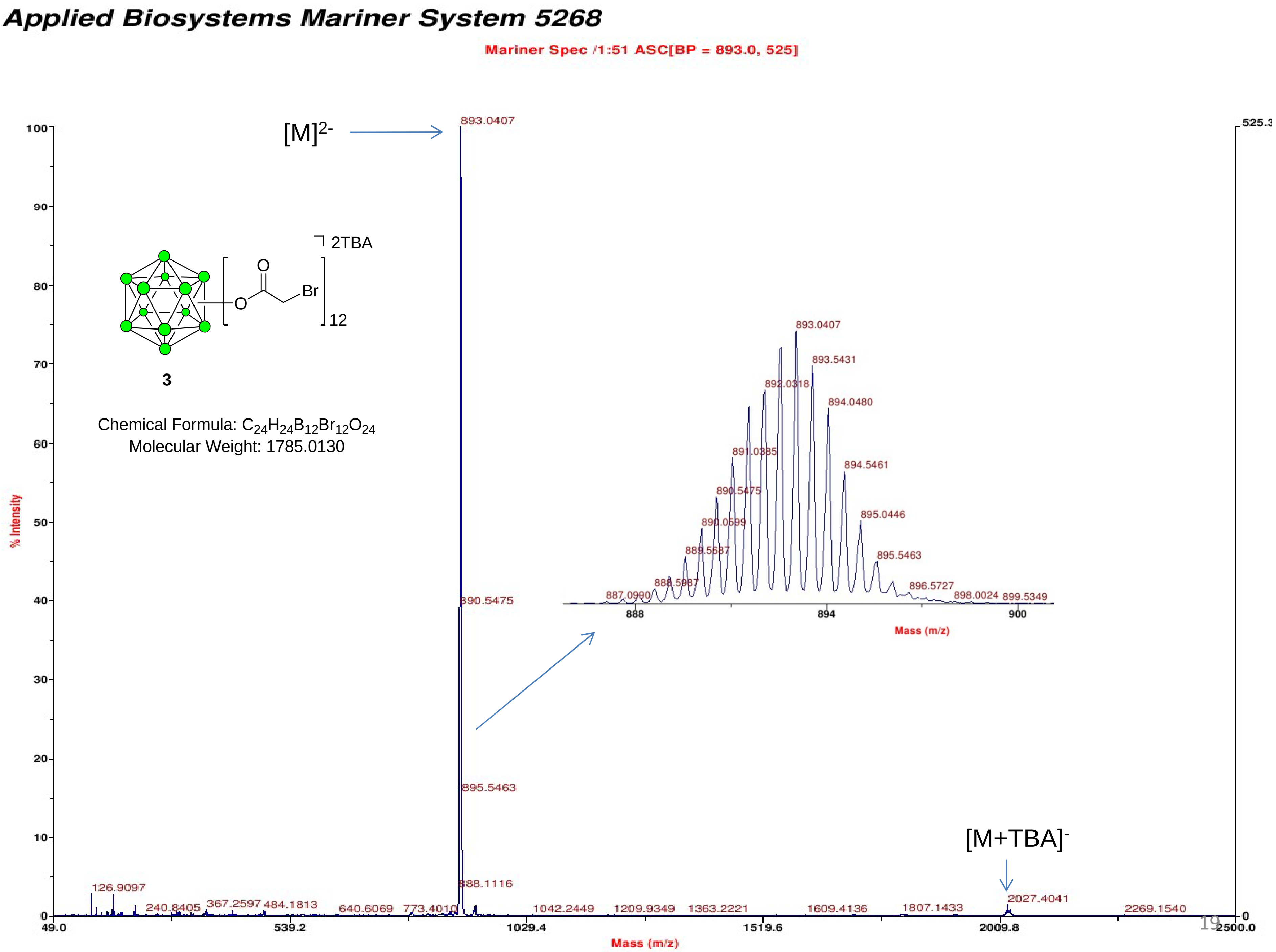

Applied Biosystems Mariner System 5268
Mariner Spec /1:51 ASC[BP = 893.0, 525]
[M]2-
893.0407
2TBA
O
Br
O
12
3
Chemical Formula: C24H24B12Br12O24
Molecular Weight: 1785.0130
% Intensity
890.5475
895.5463
888.1116
126.9097
240.8405
367.2597
484.1813
640.6069
773.4010
1042.2449
1209.9349
1363.2221
1609.4136
1807.1433
2027.4041
2269.1540
[M+TBA]-
Mass (m/z)
893.0407
893.5431
892.0318
894.0480
891.0385
894.5461
890.5475
895.0446
890.0599
889.5687
895.5463
888.5987
887.0990
896.5727
898.0024
899.5349
Mass (m/z)

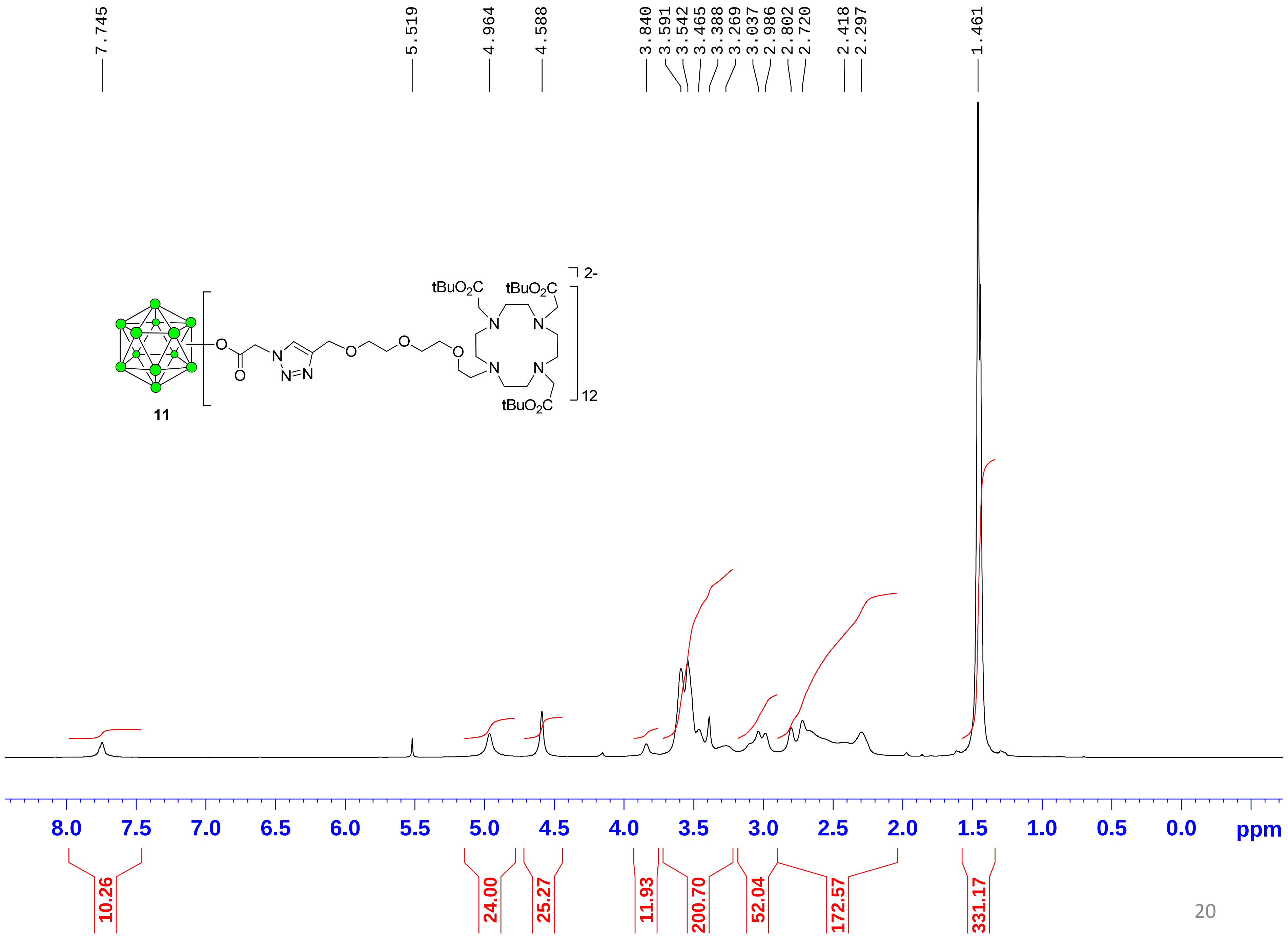

7.745
5.519
4.964
4.588
3.840
3.591
3.542
3.465
3.388
3.269
3.037
2.986
2.802
2.720
2.418
2.297
1.461
tBuO2C
tBuO2C
tBuO2C
2-
12
11
8.0 7.5 7.0 6.5 6.0 5.5 5.0 4.5 4.0 3.5 3.0 2.5 2.0 1.5 1.0 0.5 0.0 ppm
10.26
24.00
25.27
11.93
200.70
52.04
172.57
331.17

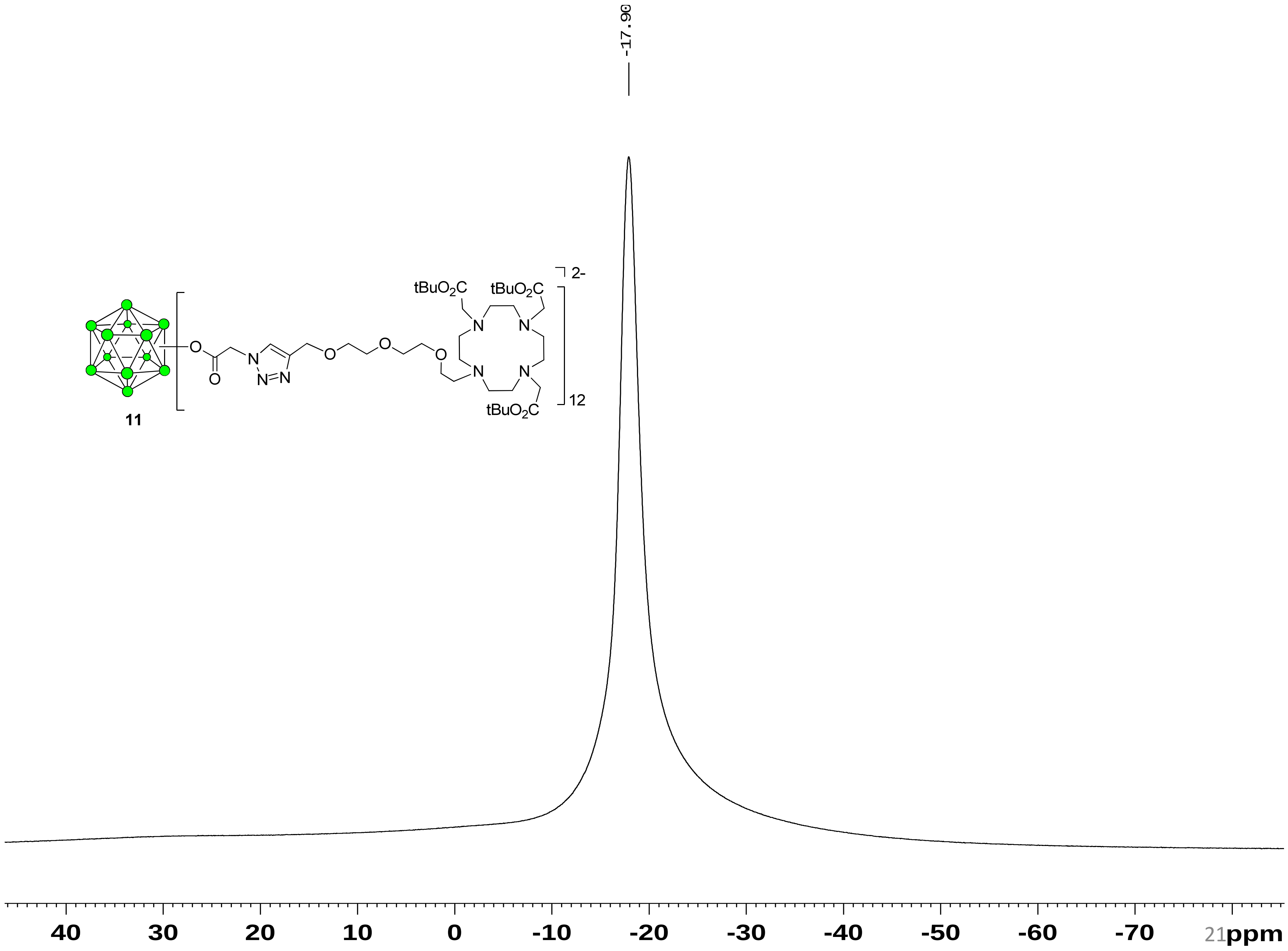
-17.90
tBuO2C
tBuO2C
2-
O
O
N
N=N
O
O
O
N
N
N
N
12
tBuO2C
11
40
30
20
10
0
-10
-20
-30
-40
-50
-60
-70
ppm

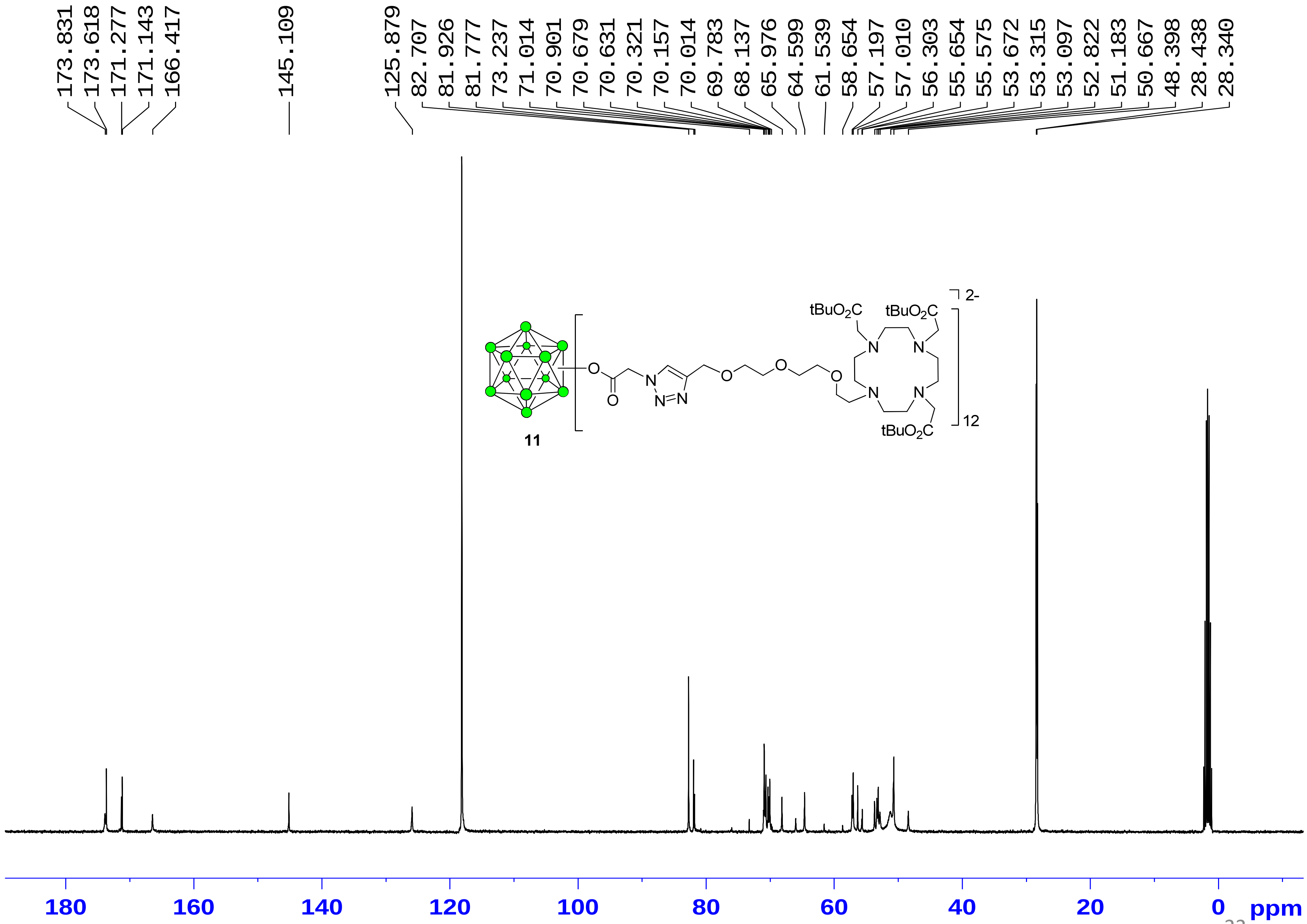
173.831
173.618
171.277
171.143
166.417
145.109
125.879
82.707
81.926
81.777
73.237
71.014
70.901
70.679
70.631
70.321
70.157
70.014
69.783
68.137
65.976
64.599
61.539
58.654
57.197
57.010
56.303
55.654
55.575
53.672
53.315
53.097
52.822
51.183
50.667
48.398
28.438
28.340
tBuO2C
tBuO2C
tBuO2C
2-
12
11
180
160
140
120
100
80
60
40
20
0
ppm

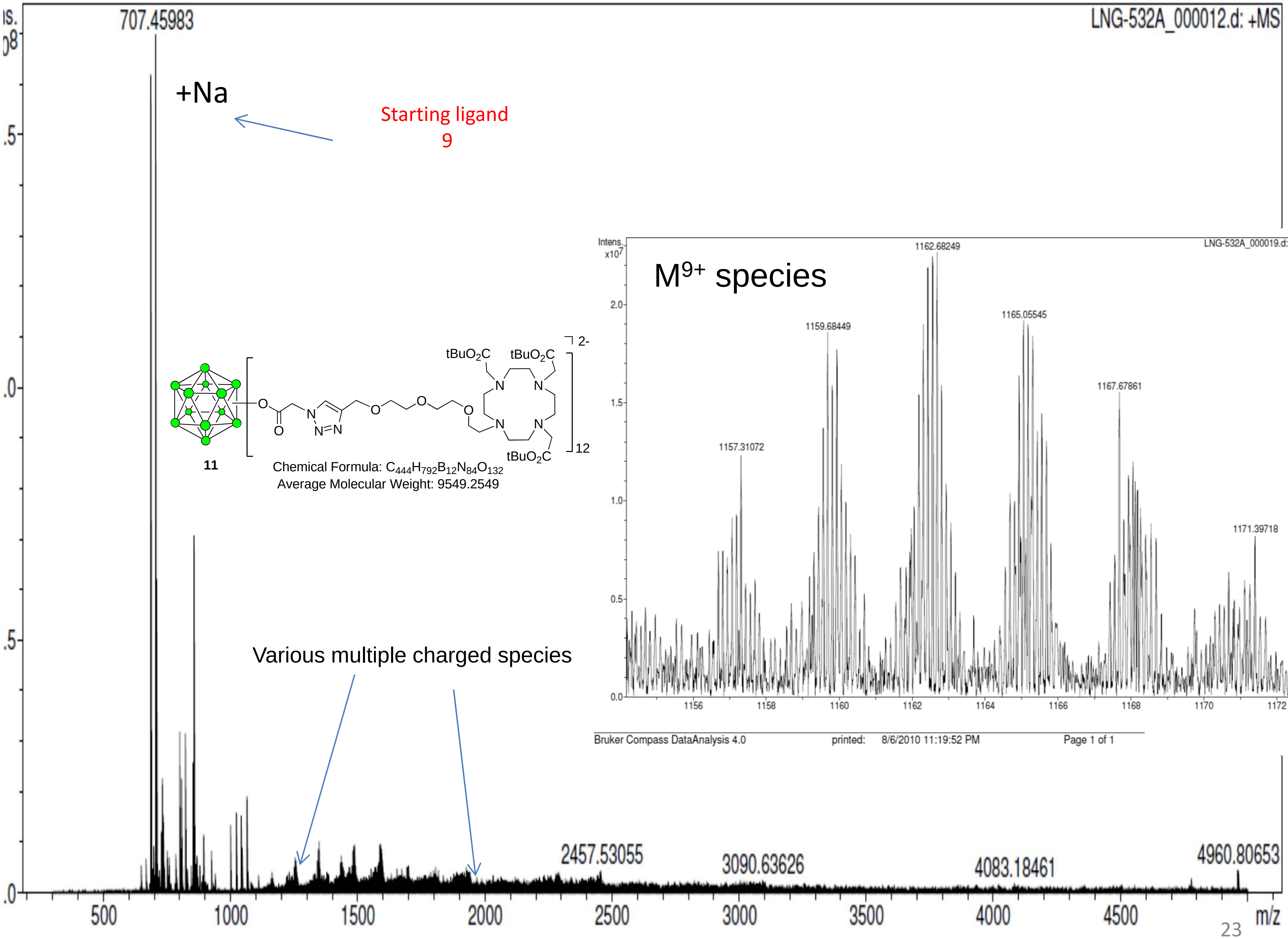
707.45983
LNG-532A_000012.d: +MS
+Na
Starting ligand
9
tBuO2C
tBuO2C
2-
12
tBuO2C
11
Chemical Formula: C444H792B12N84O132
Average Molecular Weight: 9549.2549
Intens. x10^7
LNG-532A_000019.d: +MS
M9+ species
1162.68249
1159.68449
1165.05545
1167.67861
1157.31072
1171.39718
Bruker Compass DataAnalysis 4.0
printed: 8/6/2010 11:19:52 PM
Page 1 of 1
Various multiple charged species
2457.53055
3090.63626
4083.18461
4960.80653
m/z

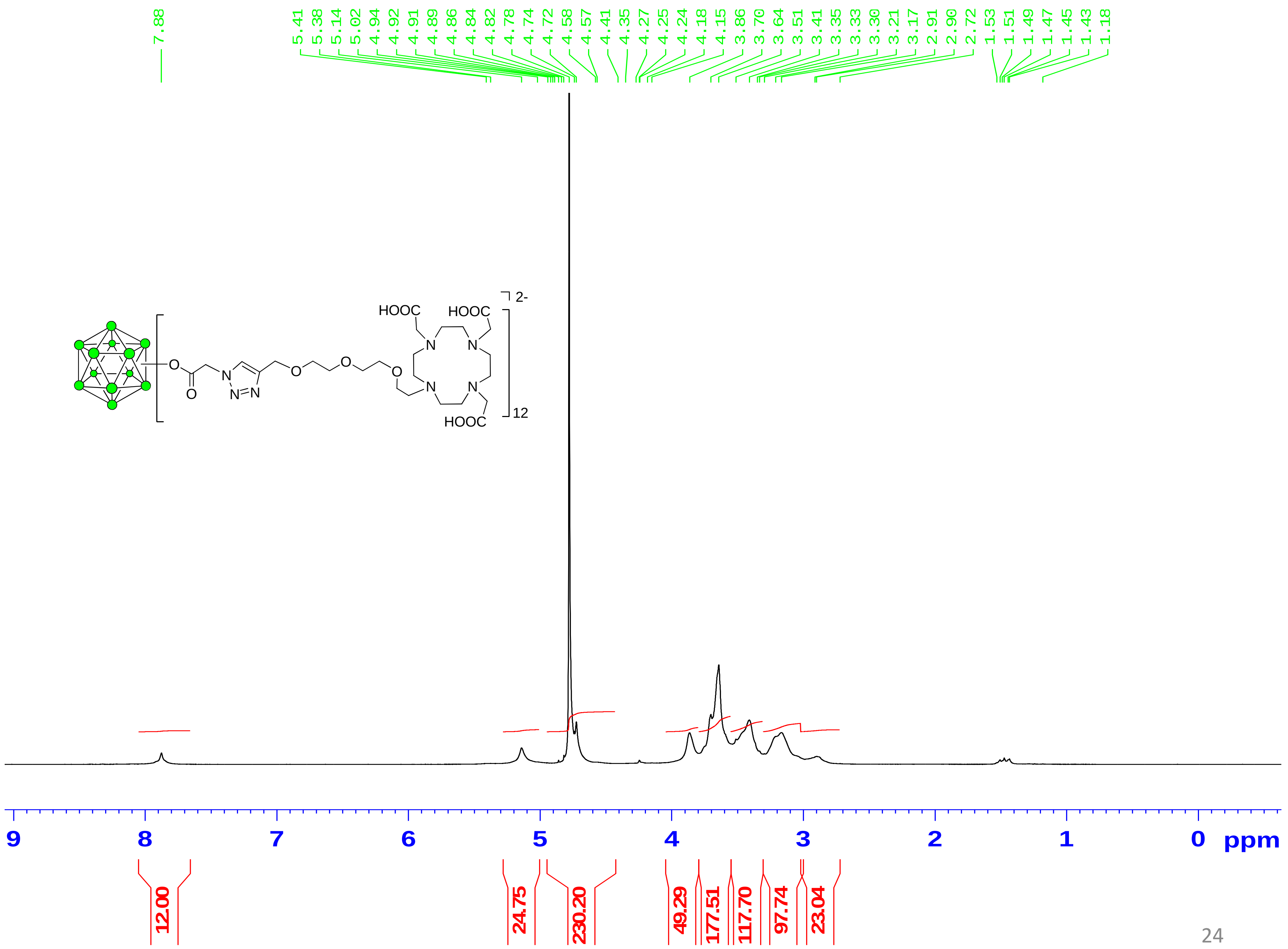
7.88
5.41 5.38 5.14 5.02 4.94 4.92 4.91 4.89 4.86 4.84 4.82 4.78 4.74 4.72 4.58 4.57 4.41 4.35 4.27 4.25 4.24 4.18 4.15 3.86 3.70 3.64 3.51 3.41 3.35 3.33 3.30 3.21 3.17 2.91 2.90 2.72 1.53 1.51 1.49 1.47 1.45 1.43 1.18
HOOC
HOOC
HOOC
2-
12
O
O
N
N=N
O
O
O
N
N
N
N
9
8
7
6
5
4
3
2
1
0
ppm
12.00
24.75
230.20
49.29
177.51
117.70
97.74
23.04

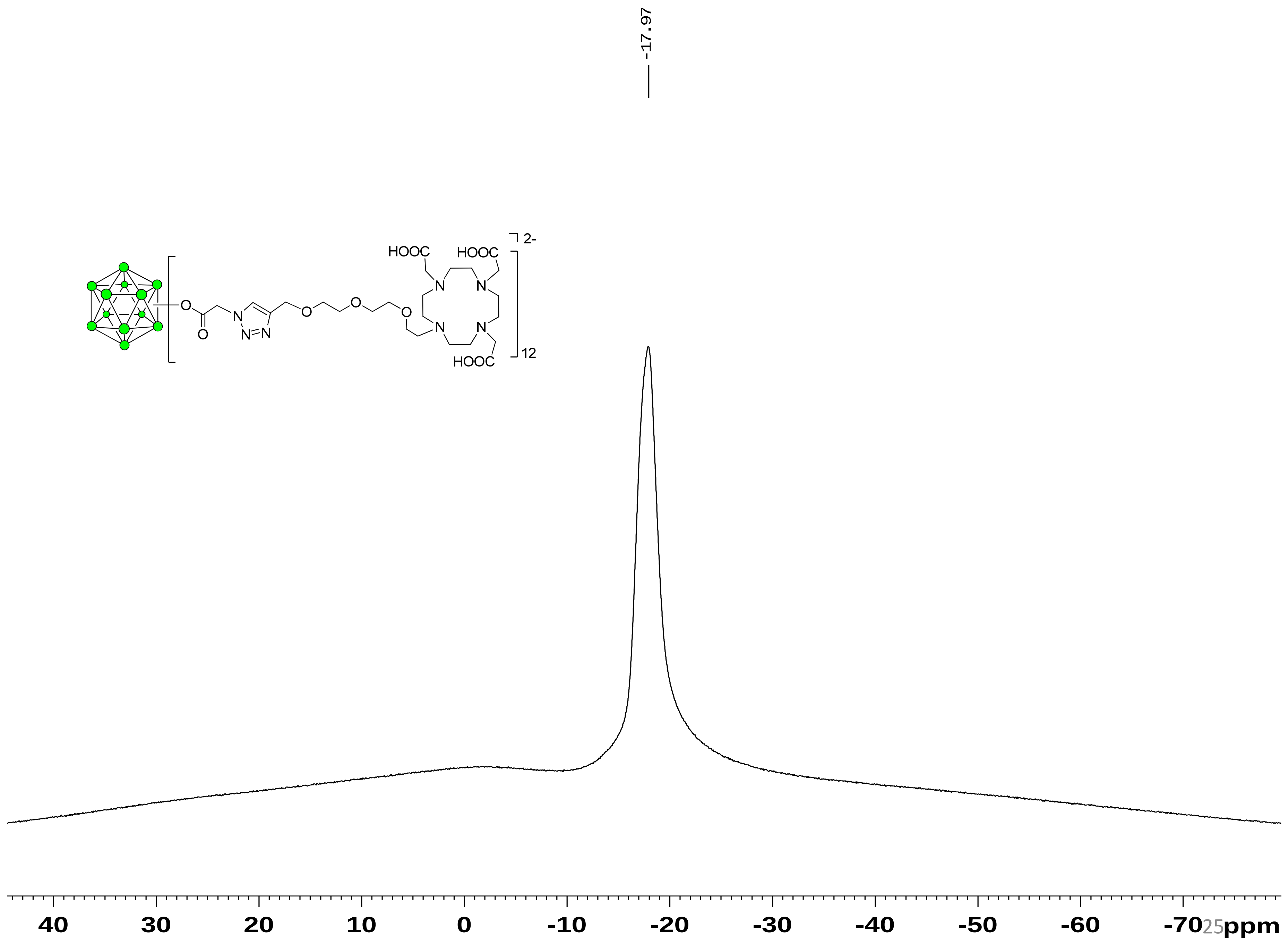
-17.97
HOOC
HOOC
HOOC
2-
12
O
O
O
O
O
N
N=N
N
N
N
N
40
30
20
10
0
-10
-20
-30
-40
-50
-60
-70
ppm

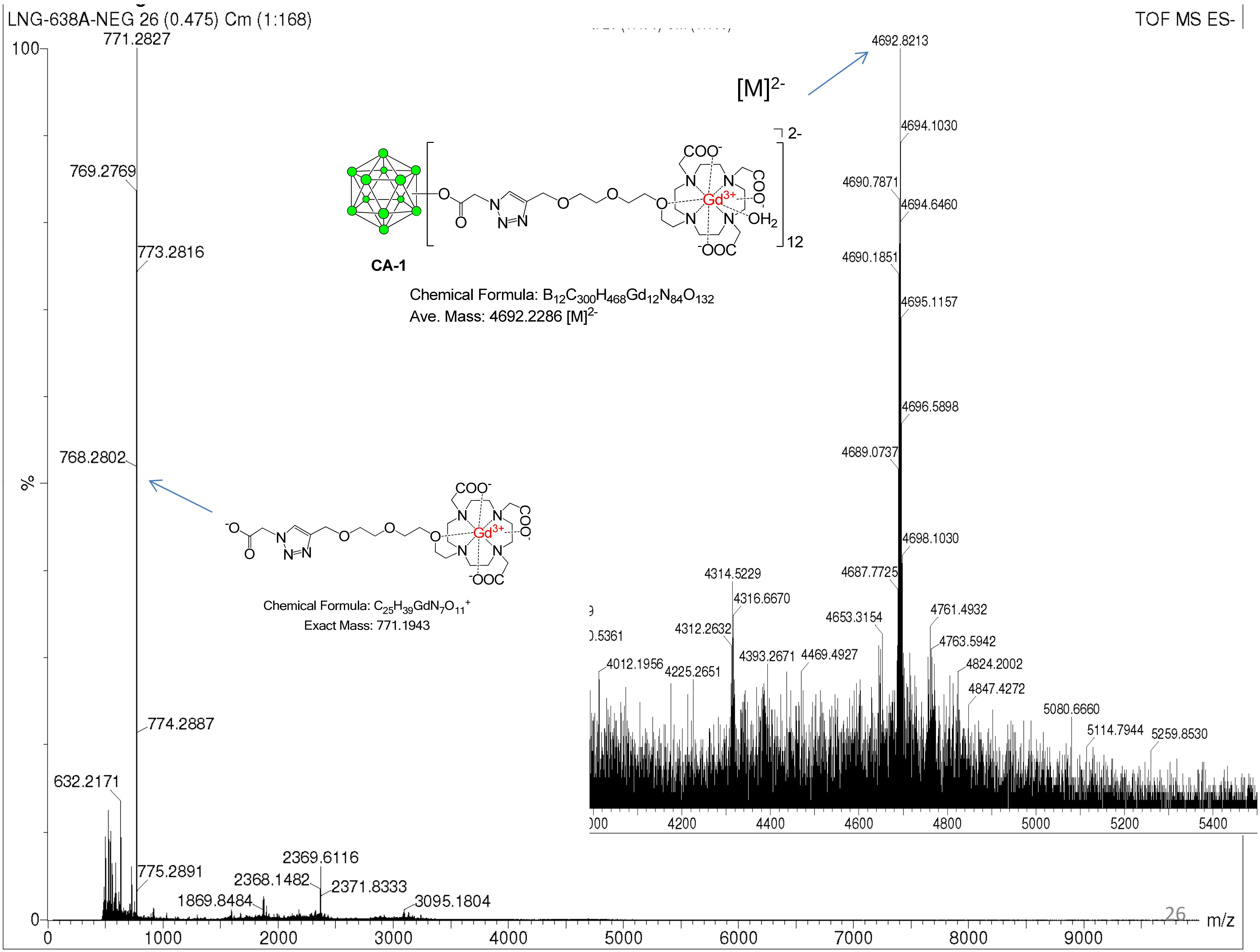

LNG-638A-NEG 26 (0.475) Cm (1:168)
TOF MS ES-
100
%
0
771.2827
769.2769
773.2816
768.2802
774.2887
632.2171
775.2891
1869.8484
2368.1482
2369.6116
2371.8333
3095.1804
0
1000
2000
3000
4000
5000
6000
7000
8000
9000
m/z
[M]2-
4692.8213
4694.1030
4690.7871
4694.6460
4690.1851
4695.1157
4696.5898
4689.0737
4698.1030
4687.7725
4314.5229
4316.6670
4653.3154
4761.4932
4312.2632
4763.5942
4012.1956
4225.2651
4393.2671
4469.4927
4824.2002
4847.4272
5080.6660
5114.7944
5259.8530
4200
4400
4600
4800
5000
5200
5400
2-
12
CA-1
Chemical Formula: B12C300H468Gd12N84O132
Ave. Mass: 4692.2286 [M]2-
Chemical Formula: C25H39GdN7O11+
Exact Mass: 771.1943

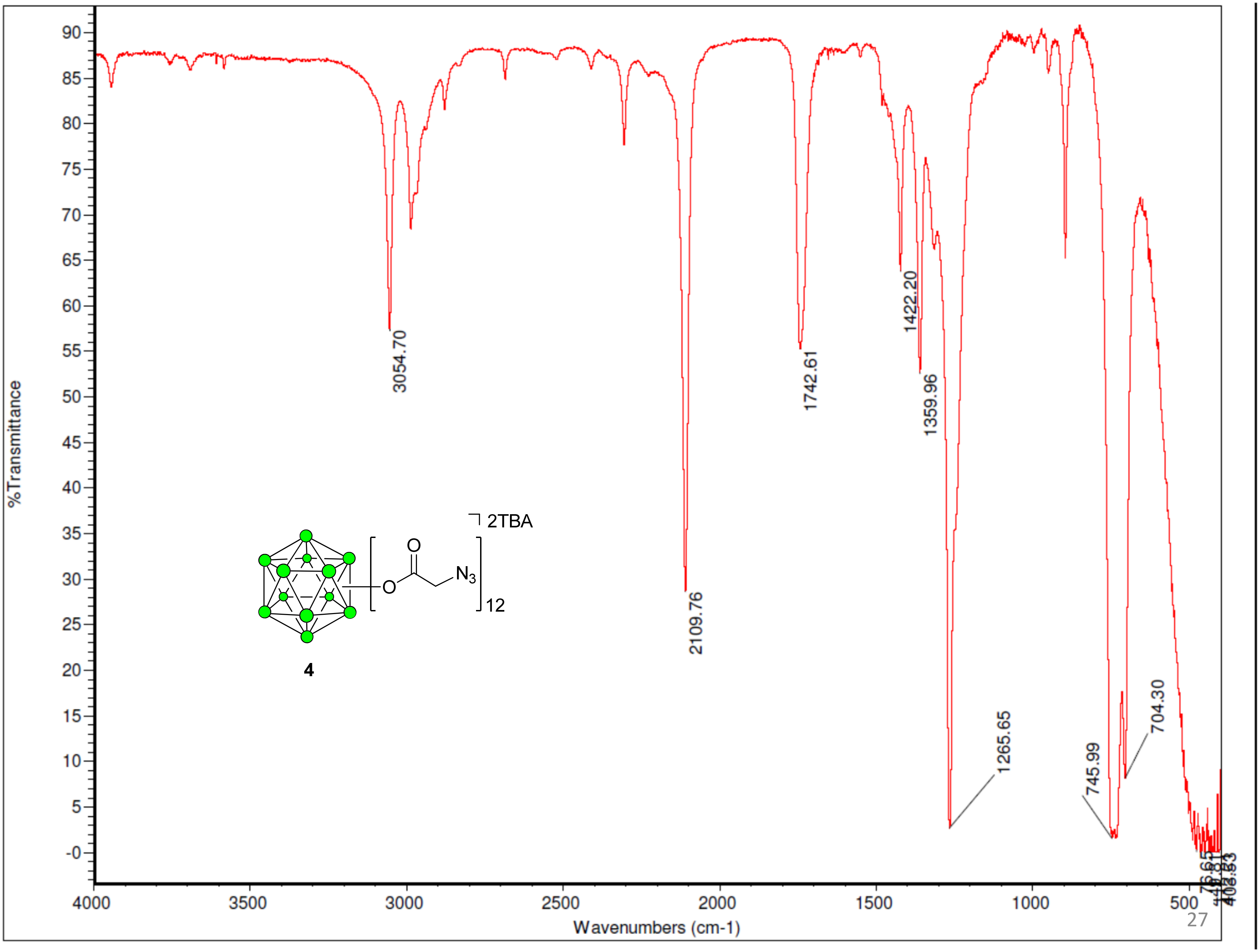
%Transmittance
Wavenumbers (cm-1)
3054.70
2109.76
1742.61
1422.20
1359.96
1265.65
745.99
704.30
2TBA
O
O
N3
12
4

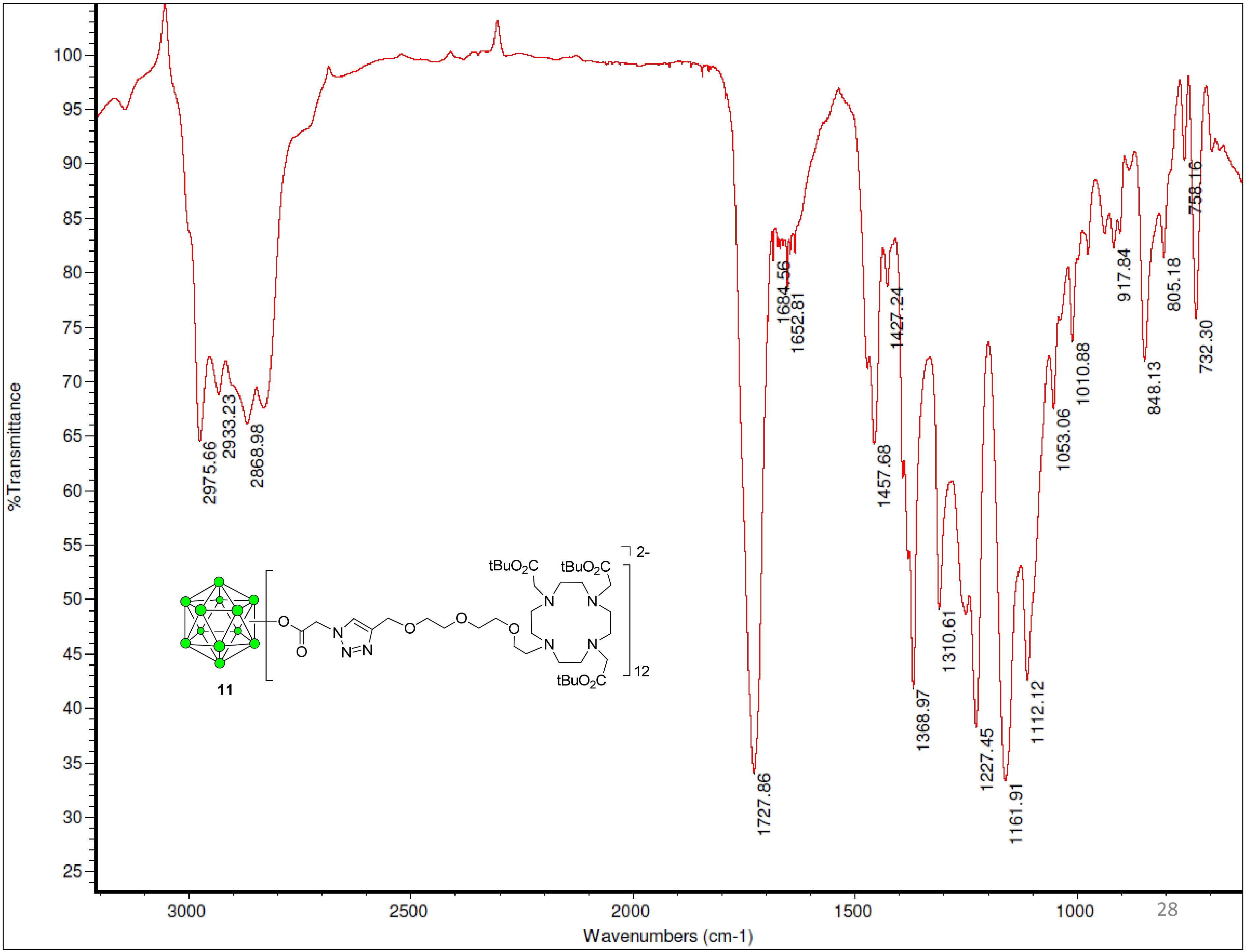
%Transmittance
Wavenumbers (cm-1)
100
95
90
85
80
75
70
65
60
55
50
45
40
35
30
25
3000
2500
2000
1500
1000
2975.66
2933.23
2868.98
1727.86
1684.56
1652.81
1457.68
1427.24
1368.97
1310.61
1227.45
1161.91
1112.12
1053.06
1010.88
917.84
848.13
805.18
758.16
732.30
tBuO2C
tBuO2C
tBuO2C
2-
12
11

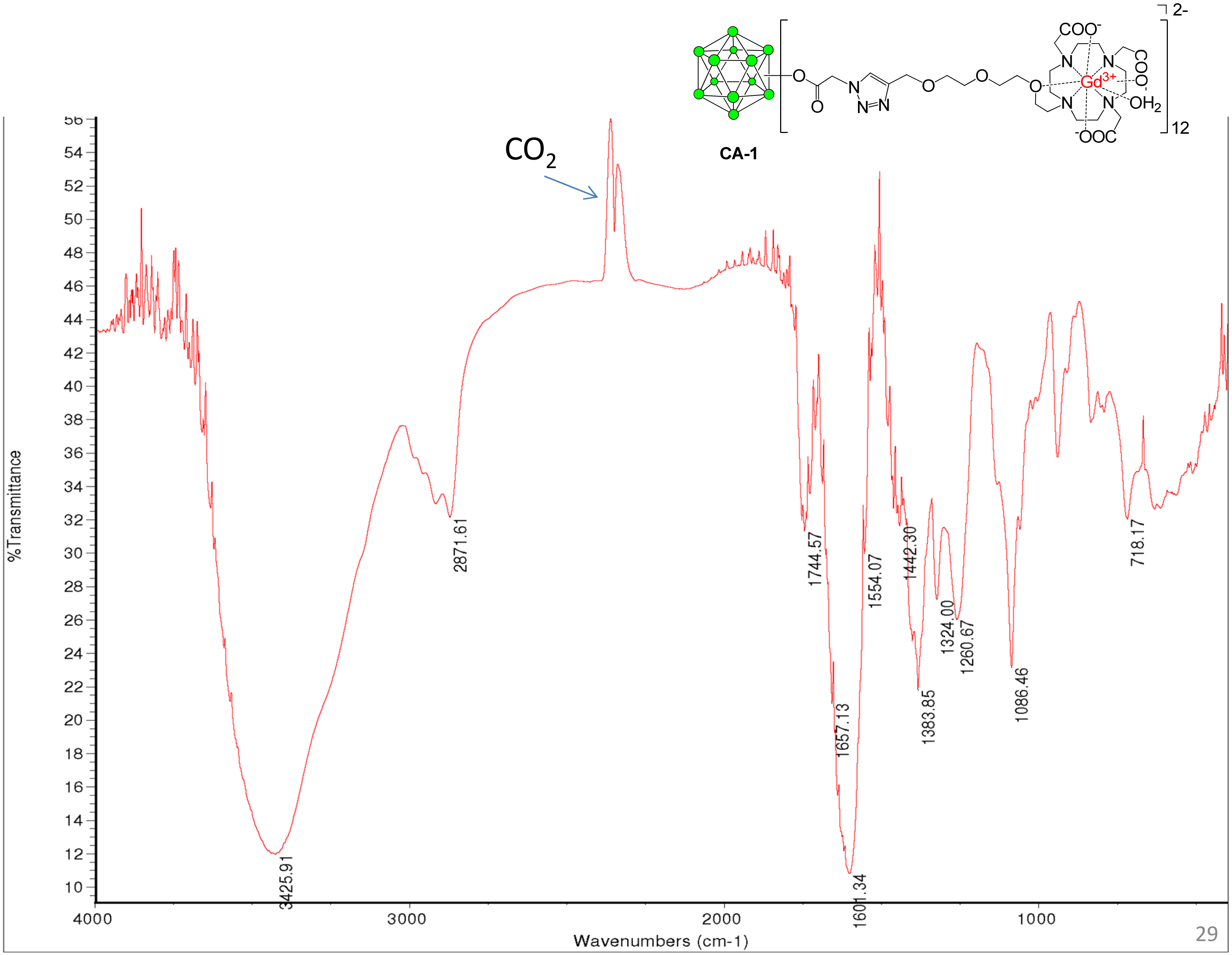
CO2
CA-1
2-
12
Gd3+
OH2
%Transmittance
Wavenumbers (cm-1)
3425.91
2871.61
1744.57
1657.13
1601.34
1554.07
1442.30
1383.85
1324.00
1260.67
1086.46
718.17

Determination of Hydration number (q) for DO3A Dy^{3+} complex 10

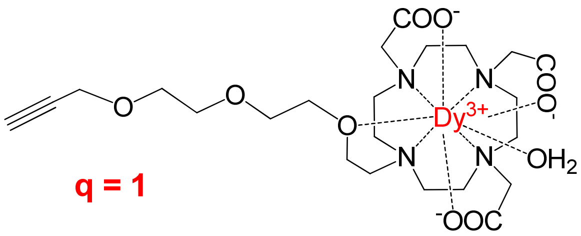

q = 1

DOTA-Dy^{3+} complex **10**

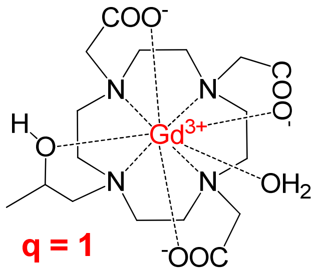

q = 1

ProHance (BARACCO)

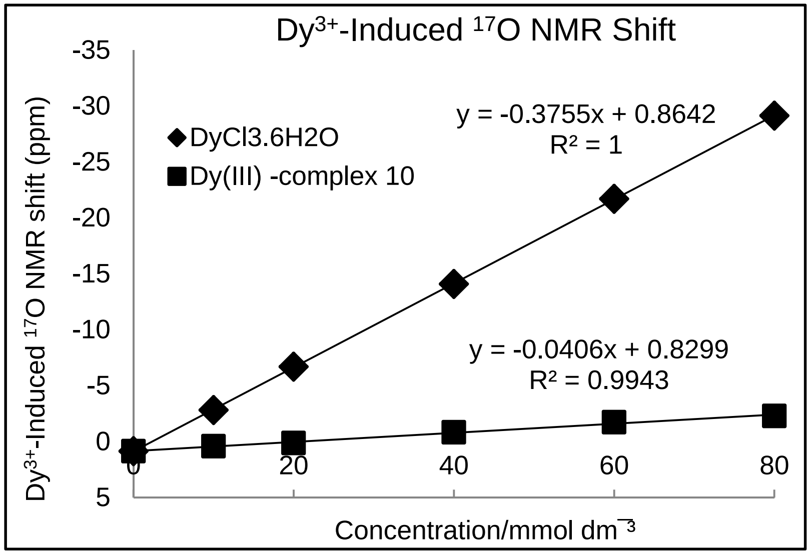

Varying concentrations of **10** and $DyCl_3.6H_2O$ over the range 10-80 mmol dm^{-3} were prepared in 80% D_2O-H_2O and the pH of the solutions was adjusted to pH 7.0. The ^{17}O NMR experiments were performed at 400 MHz using a Bruker Avance instrument at RT with the deuterium signal locked. A graph was plotted between the d.i.s. (Δδ) and the concentration for both **10** and the $DyCl_3.6H_2O$ solutions and slope was obtained. The Δδ value for a complex with the general formula, $Dy(ligand)_n(H_2O)_q$, is given by the following relation;

$$(\Delta\delta) = q\Delta[Dy(ligand)_n(H_2O)_q]/[H_2O] \qquad (I)$$

The slope of a plot of the d.i.s. versus the Dy^{3+} concentration is proportional to the **q** value of the complex (Figure). The **q** value was obtained by linearly fitting the Δδ value and was found to be 2 for complex **10**.

From the graph, which fit well to a straight line, the slope was calculated. From relation I, the slope of the graph can be equated with the following;

Slope = qΔ/[H2O],

From the graph, the slope was calculated as,

Slope = -375 ppm dm^3 mol^{-1} or,

qΔ/[H2O] = -375 ppm dm^3 mol^{-1}

For $DyCl_3$, the value of **q** was assumed to be 9, because the coordination number of Dy(III) in such complexes known to be 9. Hence, the value of Δ/[H2O] is calculated as follows:

Δ/[H2O] = -375 ppm dm^3 mol^{-1} /9 or

Δ/[H2O] = - 41.7 ppm dm^3 mol^{-1}

From this calculation, a value of q for the DOTA-Dy^{3+} complex **10** is calculated as follows:

For complex **10**, Slope = qΔ/[H2O], or

q = slope/(Δ/[H2O]) or q = -40.6/-41.7, hence

q = 1

Figure. Comparison of the per-Gd r_1 values of **CA-1**, ligand **9** and Omniscan.

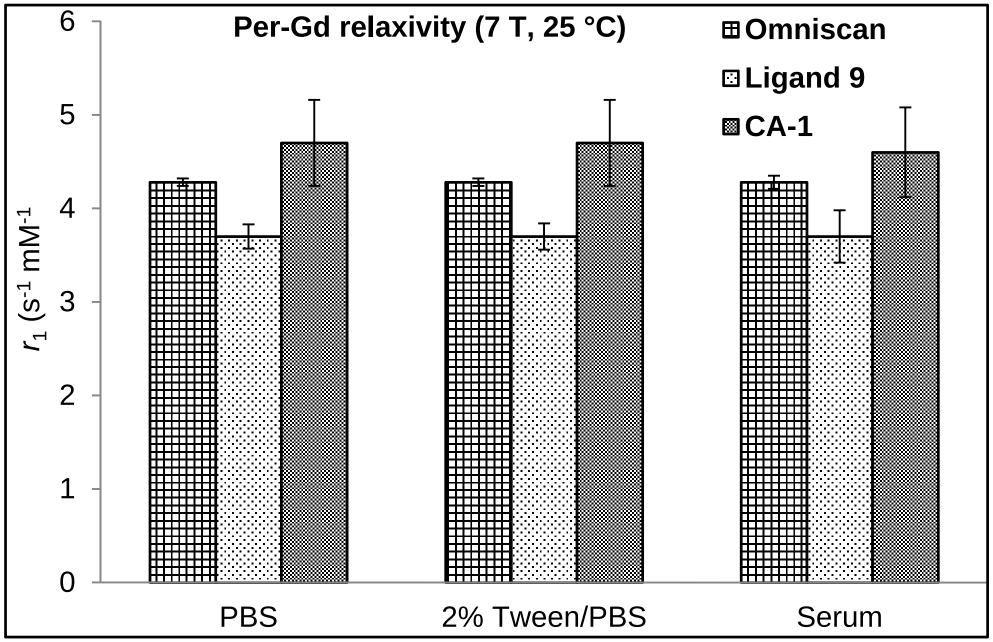

Dynamic Light Scattering Analysis of **CA-1**

1mM solutions;

Standard: Gold nanoparticles (20 nm)

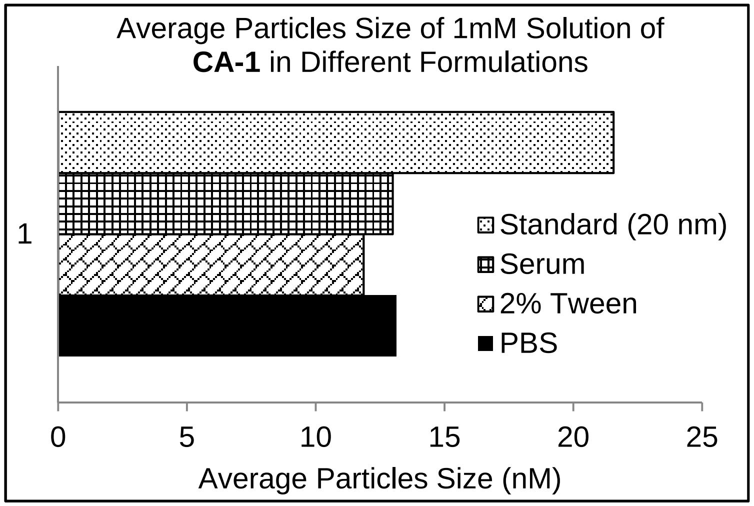

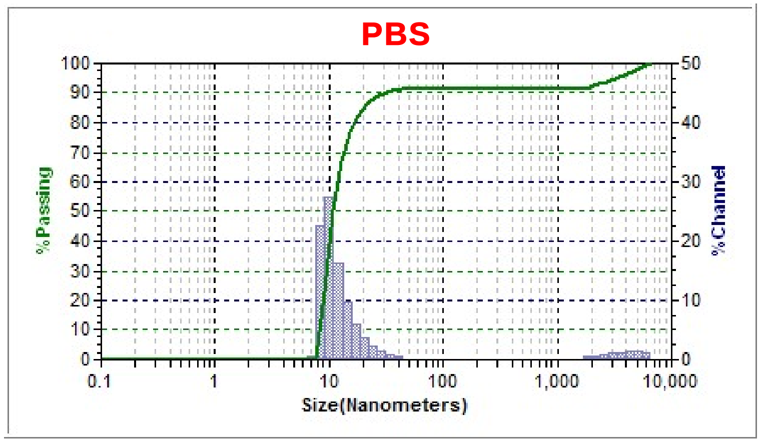

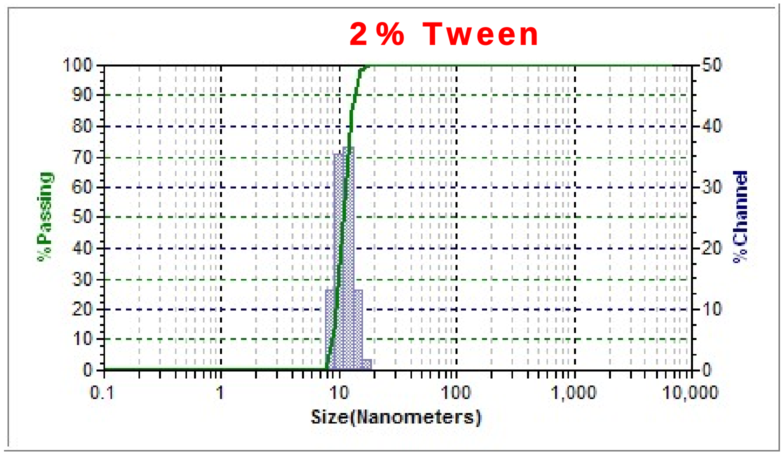

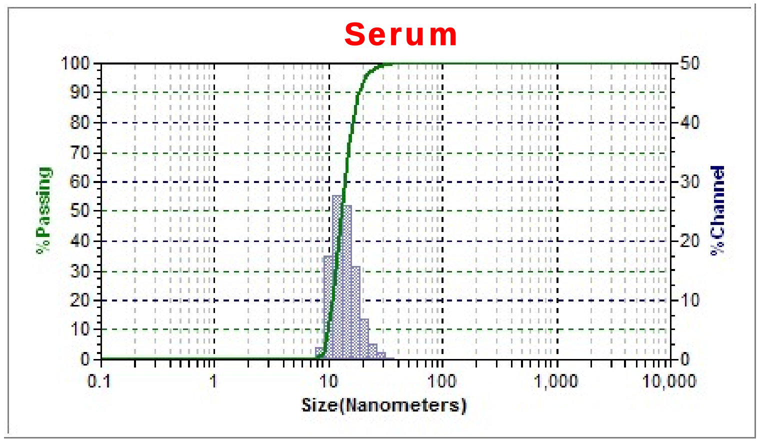

SE-HPLC analysis of CA-1

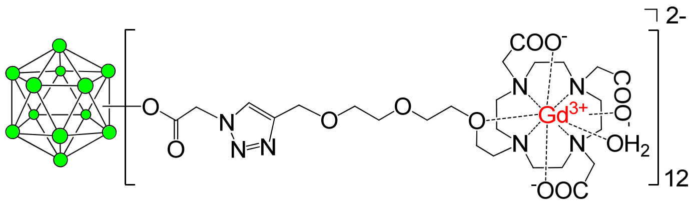

CA-1

- **Column:**
  - **BioSep-SEC-S2000 (Phenomenex)**
  - **250 X 2.0mm**
- **Mobile phase:**
  - **PBS, pH 6.6**
  - **Flow rate: 0.1 ml/min**
- **Detector:**
  - **UV-256 nm**
- **Injection:**
  - **5.0 mg/ml PBS stock solution**
  - **5ul injection**

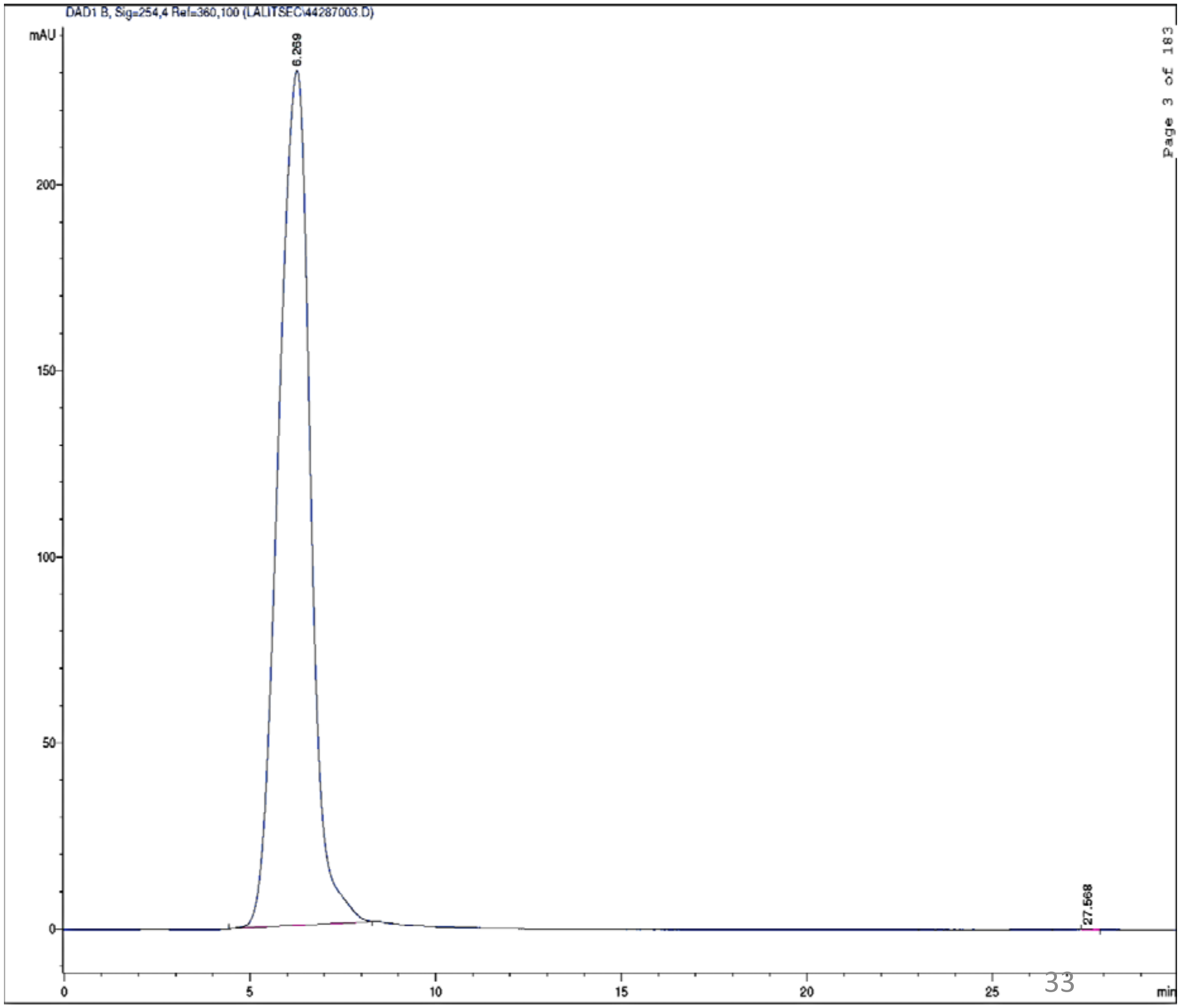

Supplement: Supplementary File 1 [file molecules-18-09034-s001.pdf]
